# A multi-mineral intervention to counter pro-inflammatory activity and to improve the barrier in human colon organoids

**DOI:** 10.3389/fcell.2023.1132905

**Published:** 2023-07-05

**Authors:** James Varani, Shannon D. McClintock, Daniyal M. Nadeem, Isabelle Harber, Dania Zeidan, Muhammad N. Aslam

**Affiliations:** Department of Pathology, The University of Michigan Medical School, Ann Arbor, MI, United States

**Keywords:** colon organoids, cytokines, gut barrier, inflammation, LPS, multi-mineral, proteomics, ulcerative colitis

## Abstract

**Introduction:** Ulcerative colitis is a chronic inflammatory condition, and continuous inflammatory stimulus may lead to barrier dysfunction. The goal of this study was to assess barrier proteomic expression by a red algae-derived multi-mineral intervention in the absence or presence of pro-inflammatory insult.

**Methods:** Human colon organoids were maintained in a control culture medium alone or exposed to lipopolysaccharide with a combination of three pro-inflammatory cytokines [tumor necrosis factor-α, interleukin-1β and interferon-γ (LPS-cytokines)] to mimic the environment in the inflamed colon. Untreated organoids and those exposed to LPS-cytokines were concomitantly treated for 14 days with a multi-mineral product (Aquamin^®^) that has previously been shown to improve barrier structure/function. The colon organoids were subjected to proteomic analysis to obtain a broad view of the protein changes induced by the two interventions alone and in combination. In parallel, confocal fluorescence microscopy, tissue cohesion and transepithelial electrical resistance (TEER) measurements were used to assess barrier structure/function.

**Results:** The LPS-cytokine mix altered the expression of multiple proteins that influence innate immunity and promote inflammation. Several of these were significantly decreased with Aquamin^®^ alone but only a modest decrease in a subset of these proteins was detected by Aquamin^®^ in the presence of LPS-cytokines. Among these, a subset of inflammation-related proteins including fibrinogen-β and -γ chains (FGB and FGG), phospholipase A2 (PLA2G2A) and SPARC was significantly downregulated in the presence of Aquamin^®^ (alone and in combination with LPS-cytokines); another subset of proteins with anti-inflammatory, antioxidant or anti-microbial activity was upregulated by Aquamin^®^ treatment. When provided alone, Aquamin^®^ strongly upregulated proteins that contribute to barrier formation and tissue strength. Concomitant treatment with LPS-cytokines did not inhibit barrier formation in response to Aquamin^®^. Confocal microscopy also displayed increased expression of desmoglein-2 (DSG2) and cadherin-17 (CDH17) with Aquamin^®^, either alone or in the presence of the pro-inflammatory stimulus. Increased cohesion and TEER with Aquamin^®^ (alone or in the presence of LPS-cytokines) indicates improved barrier function.

**Conclusion:** Taken together, these findings suggest that multi-mineral intervention (Aquamin^®^) may provide a novel approach to combating inflammation in the colon by improving barrier structure/function as well as by directly altering the expression of certain pro-inflammatory proteins.

## 1 Introduction

Ulcerative colitis (UC) is a chronic disease that manifests as diffuse mucosal inflammation and fragility of the inner lining of the large intestine (McGuckin et al., 2009; Ordás et al., 2012). The underlying pathogenesis of inflammatory bowel diseases including UC is dysregulation of the mucosal immune system and dysfunction of the epithelial barrier (Strober et al., 2007). The therapeutic armamentarium currently used for UC is aimed, primarily, at controlling inflammation (Ordás et al., 2012). Agents that target inflammation broadly including steroids and non-steroidal anti-inflammatory drugs such as mesalamine have long been treatment options. More recently, biological agents targeting specific components of the inflammatory process have become part of the treatment combination (Park and Jeen, 2015). The use of biologicals has dramatically expanded in recent years, but clinical remission rates continue to be approximately 20% over placebo in clinical trials (Feagan et al., 2013), suggesting that additional aspects of UC pathophysiology need to be addressed. In addition, current therapeutic expansion has focused mainly on addressing severe disease. There have been no therapeutic improvements to specifically address mild or less severe UC since the introduction of mesalamine in the 1980s (Hanauer et al., 1993). Mild and moderate disease represents approximately 70% of the population with UC, but few options outside of mesalamine-based treatments are available (Langholz et al., 1991; Solberg et al., 2009). Identifying ways to impact mild to moderate disease is essential.

Abnormal barrier function in the gastrointestinal tract with increased mucosal permeability is a well-described pathologic feature of UC (Salim and Söderholm, 2011; Antoni et al., 2014; Coskun, 2014; Vivinus-Nébot et al., 2014; Lee et al., 2018), but there are no therapies that directly address this fundamental disease element (Chang et al., 2017). The colonic barrier is a complex, multi-faceted entity. Tight junctional complexes, present at the apical surface of colonic epithelial cells, open and close to regulate inter-cellular transport of soluble moieties (Cereijido et al., 1998; Schmitz et al., 1999; Zeissig et al., 2007; Zihni et al., 2016). Adherens junctions are closely associated with tight junctional complexes and help organize these structures into functional units (Baumgartner, 2013). Adherens junctions and, especially, desmosomes are also largely responsible for tissue cohesion and strength (Green and Simpson, 2007; Kowalczyk and Green, 2013; Garcia et al., 2018). Effective control of permeability in the mechanically active tissue such as the gastrointestinal tract cannot be maintained if tissue strength and cohesion are compromised. An additional contributor to barrier function is the mucinous layer covering the apical surface. This layer consists of mucin proteins organized with trefoils; the mucinous layer contributes to barrier function by trapping bacteria and other particulates in the colonic stream and preventing them from reaching the epithelial surface (Pullan et al., 1994; Hensel et al., 2014).

Past work in our laboratory has shown that a red algae-derived multi-mineral product (Aquamin^®^) increases the elaboration of numerous proteins that contribute to barrier structure. These studies have been conducted using human colon tissue in organoid culture (Attili et al., 2019; Aslam et al., 2020a; McClintock et al., 2020) and validated in a small interventional trial with healthy adult subjects (Aslam et al., 2021). In the organoid culture studies (using colon tissue from healthy individuals or lesional tissue from individuals with UC), we demonstrated modest changes in tight junctional protein expression with the multi-mineral intervention but much stronger upregulation of several cadherins and desmosomal proteins. Desmosome numbers per unit length were, themselves, increased with multi-mineral treatment (Attili et al., 2019; Aslam et al., 2020a). Also, strongly upregulated were mucin proteins, trefoils and members of the carcinoembryonic antigen cell adhesion molecule (CEACAM) family (Attili et al., 2019; Aslam et al., 2020a). Improved permeability control and increased tissue strength accompanied mineral-induced changes in barrier protein expression (McClintock et al., 2020). These findings highlight the importance of inorganic minerals to barrier formation in the gastrointestinal tract. They do not, however, indicate whether an effective barrier can be maintained in the face of an inflammatory attack.

As a way to begin addressing this issue, human colon organoids were exposed to lipopolysaccharide (LPS) in combination with three pro-inflammatory cytokines (LPS-cytokines) continually for a 14-day period in order to mimic the environment in the chronically inflamed colon (Putt et al., 2017; Friedrich et al., 2019). Untreated organoids and those exposed to the pro-inflammatory stimulus were concomitantly treated with the same multi-mineral intervention as used previously or kept as control. The colon organoids were then subjected to proteomic analysis and confocal fluorescence microscopy along with barrier function assays—i.e., transepithelial electrical resistance (TEER) and tissue cohesion measurements—to directly assess the extent to which multi-mineral intervention can protect against barrier loss resulting from the inflammatory stimulus. To summarize the results from the current study, the pro-inflammatory mix (LPS-cytokines) increased the expression of multiple proteins that influence innate immunity, promote inflammation and disrupt barrier. However, when the multi-mineral intervention was concomitantly present with the LPS-cytokine mix, proteins that contribute to barrier formation were still upregulated (as they were in the presence of the multi-mineral intervention alone). The reduction in barrier function was prevented.

## 2 Materials and methods

### 2.1 LPS-cytokine mix

A combination of lipopolysaccharide (LPS) from *Escherichia coli* (*E. coli*) and three pro-inflammatory cytokines was used to mimic the environment in the chronically-inflamed colon (Putt et al., 2017). The stock solution for the mix consisted of LPS (1 μg/mL; Sigma), tumor necrosis factor-α (TNF-α; 50 ng/mL, Sigma), interleukin 1-β (IL-1β; 25 ng/mL, Shenandoah Biotech), and interferon-γ (IFN-γ; 50 ng/mL, Sigma). The same combination (in the same concentrations) was also utilized by Putt et al. (2017). For the current study, the pro-inflammatory mix was initially assessed over a wide range of concentrations for organoid toxicity at day-14 with cultures established from two subjects. Toxicity was defined based on morphological evidence of organoid growth suppression (evident by the presence of smaller organoids), organoid failure to demonstrate features of differentiation (formation of thick walls and decreased budding structures) and loss of tissue integrity (Supplementary Figure S1). High concentrations (≤1:50 dilution) of the pro-inflammatory mix stock solution were toxic, based on these criteria. Findings were similar with organoids from either source. Based on these preliminary studies, subsequent experiments were carried out using a 1:250 dilution of the LPS-cytokines stock solution, providing 4 ng/mL of LPS. A similar dose of LPS has been used by others in *ex vivo* studies (Guo et al., 2013).

### 2.2 Aquamin^®^

Aquamin^®^ is a calcium-, magnesium- and multi-trace element-rich product harvested from the skeletal remains of the red marine algae, Lithothamnion sp (Adey and McKibbin, 1970) (Marigot Ltd., Cork, Ireland). The calcium and magnesium ratio in Aquamin^®^ is approximately (12:1); Aquamin^®^ also contains measurable levels of seventy-two trace minerals. Mineral composition of Aquamin^®^ was established via an independent laboratory (Advanced Laboratories; Salt Lake City, Utah) using Inductively Coupled Plasma Optical Emission Spectrometry. Supplementary Table S1 is a list of elements detected in Aquamin^®^ and their relative amounts. Aquamin^®^ is sold as a dietary supplement (GRAS 000028) and is used in various products for human consumption in Europe, Asia, Australia, and North America. In the *ex vivo* studies reported on here, Aquamin^®^ was used at an amount (of 0.925 mg/mL) providing a final calcium concentration of 3 mM. This concentration was chosen based on earlier studies (Attili et al., 2019; Aslam et al., 2020a; McClintock et al., 2020) demonstrating increases in multiple cell-cell adhesion proteins including several desmogleins along with an increase in desmosomes as evidenced by transmission electron microscopy (Attili et al., 2019; Aslam et al., 2020a).

### 2.3 Colon organoid culture

Normal-appearing tissue obtained endoscopically from the sigmoid colon from three subjects was utilized (under protocol HUM00076276). The collection and use of human colonic tissue was approved by the Institutional Review Board (IRBMED) at the University of Michigan. All subjects provided written informed consent prior to colonoscopy and colon biopsy collection procedure. All procedures to obtain human tissues were conducted according to the principles stated in the Declaration of Helsinki. Additional organoids used for the transwell membrane cultures were acquired from normal colonic tissues and collected from deceased donors through the Gift of Life, Michigan (under protocol HUM00105750). Demographic characteristics (age, gender, ethnicity, and site) of subjects providing tissue are present in Supplementary Table S2.

Isolation of human colonic crypts, along with organoid cultivation was performed according to a previously described protocol (Miyoshi and Stappenbeck, 2013; Dame et al., 2014; Dame et al., 2018; McClintock et al., 2018). For the present work, cryopreserved samples from previous studies (Dame et al., 2018; Attili et al., 2019; Aslam et al., 2020a; McClintock et al., 2020) were cultured in Matrigel (354234; Corning; used at 8 mg/mL concentration). Cryopreserved cells were thawed swiftly in a 37°C water bath (less than 2 min) and then washed in complete medium two times to remove DMSO. Washed organoids were resuspended in 70 μL growth medium (see below), mixed with Matrigel (180 µL) and plated in a well of 6-well plate. Plated Matrigel was allowed to polymerize for 10 min at 37°C and then additional media (2.5 mL) was added to the wells (of six-well plate). The usual cell aggregates (organoids) to Matrigel ratio is around 10%–15% at the time of seeding in the Matrigel. These samples were expanded over a 3–4-week period. During the expansion phase, culture medium consisted of a 1:1 mix of Advanced DMEM/F12 (Invitrogen) and the same medium previously conditioned by the growth of L cells genetically modified to produce recombinant forms of Wnt3a, R-spondin-3 and Noggin (i.e., L-WRN) (Miyoshi and Stappenbeck, 2013). The growth medium also contained 10% fetal bovine serum (Gibco) and the final calcium concentration was 1.0 mM. The medium was supplemented with 1X N2 (Invitrogen), 1X B-27 without vitamin A (Invitrogen), 1 mM N-Acetyl-L-cysteine (Sigma), 10 mM HEPES (Invitrogen), 2 mM Glutamax (Invitrogen), 100 μg/mL Primocin (InvivoGen) and small molecule inhibitors: 10 μM Y27632 (Tocris); as a ROCK inhibitor, 500 nM A83-01 (Tocris); a TGF-β inhibitor, 10 μM SB202190 (Sigma); a p38 inhibitor, along with 100 ng/mL EGF (R&D). For the first 10 days of expansion of culture, and for 2 days at each passage the medium was also supplemented with 2.5 µM CHIR99021 (Tocris) to promote growth. CHIR99021 is a glycogen synthase kinase (GSK) 3 inhibitor that also acts as a Wnt activator (Miyoshi and Stappenbeck, 2013; Attili et al., 2019). During the expansion phase, the colon organoids were passaged 3–6 times in Matrigel prior to treatment with the experimental interventions.

During the experimental phase (a 14-day in-life portion of the study), established organoids were interrogated in L-WRN plus 10 µM Y27632 (but without the additional small molecules) diluted 1:4 with KGM Gold. KGM-Gold is a serum-free, calcium-free culture medium optimized for epithelial cell growth (Lonza). The final serum concentration in the L-WRN–KGM Gold culture medium (control medium) was 2.5% and the calcium concentration was 0.25 mM. This control treatment medium was compared to the same medium supplemented with the pro-inflammatory (LPS-cytokine) mix at a 1:250 dilution of the stock material. Treatment with Aquamin^®^ was concurrent with the LPS-cytokine exposure. Control organoid cultures and those exposed to the pro-inflammatory mix were maintained without additional treatment or treated with Aquamin^®^ in an amount to provide a final calcium concentration of 3.0 mM. Organoids (50–100 per condition) were evaluated by phase-contrast microscopy (Hoffman Modulation Contrast—Olympus IX70 with a DP71 digital camera) for change in size and shape during the 14-day in-life portion of the study and at harvest (described in Attili et al., 2019; McClintock et al., 2020). These organoids were then assessed for tissue cohesion. For that purpose, organoids were removed from the Matrigel, and fragmented by applying mechanical force by pipetting the entire pellet 30 times through an uncut 200 µL pipet tip. The fragments were washed three times in PBS and cultured in fresh Matrigel and treated with the same respective treatments. After 24 h, these organoids were assessed under phase-contrast microscopy at 40X (power field) and sized (*n* = 10–12 power fields at 40X per condition). Phase-contrast images were analyzed using area measurements in Adobe Photoshop (CC version 19.1.5). Average organoid size reduction was established by dividing the average pre-fragmentation surface area to the average post-fragmentation area.

At harvest, organoids were prepared for either proteomic assessment or for confocal microscopy as described below.

### 2.4 Organoid culture on transwell filters

For transwell experiments, colon organoids were dissociated into small cell aggregates (less than 40 µm in size) and plated onto collagen IV (Sigma)-coated transwells (0.4 µm pore size, 0.33 cm^2^, PET, Corning Costar) at 200,000 cell aggregates to obtain organoid-derived monolayers as described previously (McClintock et al., 2020). Cell aggregates were seeded for attachment and initial growth in growth medium (as above). The medium was also supplemented with 10 nM Gastrin (Sigma), 50 ng/mL Noggin (R&D), 50 ng/mL EGF, and 2.5 μM Y27632. After 2 days, Y27632 was removed. After 24 h, growth medium was replaced with L-WRN–KGM Gold (control medium) or with the same control medium along with the LPS-cytokine mix alone, Aquamin^®^ alone or the combination of the two. Fresh culture medium and respective treatments were added every 2 days during the incubation period. TEER values were determined daily (up to 5 days) to confirm establishment of monolayer with an Epithelial Volt Ohm Meter 2 (EVOM2; World Precision Instruments) and STX2 series chopstick electrodes.

### 2.5 Confocal fluorescence microscopy

Once the TEER assay was completed, membranes were prepared for confocal fluorescence microscopy, as previously reported (McClintock et al., 2020). The membranes were fixed for 15 min at −20°C in 100% methanol. After fixation, membranes were washed three times in PBS before blocking in 3% BSA (Sigma) in PBS for 1 h. Following this, membranes were stained with antibodies to occludin (331594; Invitrogen; 1:400), desmoglein-2 (53-9159-80; eBioscience; 1:200) and cadherin-17 (NBP2-12065AF488; Novus Biologicals; 1:200) for 1 h in 1% BSA in PBS. Stained membranes were rinsed three times (5 min each) in PBS, stained with DAPI for 5 min to detect nuclei and washed with PBS three more times. Lastly, the membranes were gently cut from the transwell insert and mounted apical side up on Superfrost Plus glass slides (Fisher Scientific, Pittsburgh, PA) with Prolong Gold (P36930; Life Technologies Molecular Probes). The stained specimens were visualized and imaged with a Leica Stellaris, an inverted confocal microscope system (at the Microscopy and Imaging Laboratory, University of Michigan Medical School Biomedical Research Core Facility). At the end, ImageJ1.52n was used to quantify the intensity of the confocal microscopy-generated images for all three stains (occludin, desmoglein-2 and cadherin-17). For image analysis in ImageJ, confocal images were collected at the same laser intensities for the purposes of direct comparison. Briefly, a macro was created that would create a rectangular box roughly one-ninth of the total image size. This box was used to grid off the image into nine equal area regions in which the Mean Gray Value (MGV) was measured, with the MGV representing mean fluorescent intensity. The MGV is measured on a scale of 0–85 with 0 being completely black and 85 being the highest intensity value. Confocal-generated Z-stacks were rendered as a 3D (3-dimensional) animation movie using Fiji (ImageJ1.52n with Bio-Formats Importer plugin).

### 2.6 Differential proteomic analysis

Colon organoids were harvested from two wells per condition of a 6-well plate for proteomics by individual experiments and from each subject separately. These organoids were isolated from Matrigel using 2 mM EDTA (E-5134; Sigma) in DPBS (14190-144; Gibco) for 15 min and then exposed to Radioimmunoprecipitation assay (RIPA)—lysis and extraction buffer (Pierce, # 89901; ThermoFisher Scientific) for protein isolation at 4°C (or on ice) and by centrifugation (3 min; 100 × g; 4°C) as described in our previous reports (McClintock et al., 2018; Attili et al., 2019; Aslam et al., 2020a). Proteomic assessments were conducted by using mass spectrometry-based Tandem Mass Tagging system (TMT, ThermoFisher Scientific) in the Proteomics Resource Facility (PRF) housed in the Department of Pathology at the University of Michigan. For this, four subjects were assessed individually using TMT six-plex kits. The first experiment was conducted as part of a range-finding study with LPS-cytokines alone, and compared to control medium (L-WRN—KGM Gold culture medium). Three subsequent proteomic experiments were conducted with complete sets of samples; control medium alone, addition of LPS-cytokines or supplementing Aquamin^®^ to control medium and combination of Aquamin^®^ and LPS-cytokines.

Fifty micrograms (at a concentration of 1 μg/μL) of organoid protein from each condition was digested separately with trypsin and individual samples labeled with one of six isobaric mass tags according to the manufacturer’s protocol. After labeling, equal amounts of peptides from each condition were mixed together. In order to achieve in-depth characterization of the proteome, the labeled peptides were fractionated using 2D-LC (basic pH reverse-phase separation followed by acidic pH reverse phase) and analyzed on a high-resolution, tribrid mass spectrometer (Orbitrap Fusion Tribrid, ThermoFisher Scientific) using conditions optimized at the PRF. MultiNotch MS3 analysis was employed to obtain accurate quantitation of the identified proteins/peptides (McAlister et al., 2014). Data analysis was performed using Proteome Discoverer (v2.4, ThermoFisher Scientific). MS2 spectra were searched against UniProt human protein database (20,353 sequences; downloaded on 06/20/2019) using the following search parameters: MS1 and MS2 tolerance were set to 10 ppm and 0.6 Da, respectively; carbamidomethylation of cysteines (57.02146 Da) and TMT labeling of lysine and N-termini of peptides (229.16293 Da) were considered static modifications; oxidation of methionine (15.9949 Da) and deamidation of asparagine and glutamine (0.98401 Da) were considered variable. Identified proteins and peptides were filtered to retain only those that passed ≤2% false discovery rate (FDR) threshold of detection. Quantitation was performed using high-quality MS3 spectra (Average signal-to-noise ratio of 6 and <40% isolation interference). Proteins names were retrieved using Uniprot.org, and Reactome v82 (reactome.org) was used for pathway enrichment analyses (Gillespie et al., 2022). Only Proteins with a ≤2% FDR confidence of detection were included in the analyses. GeneCodis v4 was used for additional functional enrichment analyses to characterize responses (Garcia-Moreno et al., 2022). GeneCodis analysis provides information taken from Gene Ontology (GO) related to cellular components, molecular functions, and biological processes. In addition to Reactome, it also presents Panther Pathways, WikiPathways and Kyoto Encyclopedia of Genes and Genomes (KEGG) pathways data.

Differential protein expression profiling (for each subject separately) was established by considering the respective control (L-WRN–KGM Gold medium) as a comparator and evaluated results from the other conditions in relation to this control. Next, resulting data from the three datasets were combined. The initial analysis was based on an unbiased proteome-wide screen of all proteins modified by LPS-cytokines and Aquamin^®^ interventions in relation to the control using a cutoff of 1.8-fold as used previously (McClintock et al., 2018; Attili et al., 2019; Aslam et al., 2020a). Follow-up analysis involved a targeted approach towards inflammation, differentiation, barrier-related, cell-cell and cell adhesion proteins. The mass spectrometry proteomics data were deposited to the ProteomeXchange Consortium via the PRIDE partner repository (with the dataset identifier PXD038941) for open access.

### 2.7 Statistical analysis

Means and standard deviations were obtained for individual proteins, pre- and post-split size, TEER and confocal intensity measurements. Groups were analyzed by one-way analysis of variance (ANOVA) followed by unpaired *t*-test (two-tailed) using GraphPad Prism (v 9.1). Pathways enrichment data reflect Reactome-generated *p*-values based on the number of entities identified in a given pathway as compared to total proteins involved in that pathway. A binomial test was applied within Reactome to calculate the probability shown for each result, and the *p*-values were corrected for the multiple testing (Benjamini–Hochberg procedure) that arises from evaluating the submitted list of identifiers against every pathway. For GeneCodis enrichment analysis, the complete set of annotated proteins generated (our own reference set) was used as background. The hypergeometric or Wallenius test generated raw *p*-values and correction was applied via Benjamini/Hochberg FDR. Data were considered significant at *p* < 0.05.

## 3 Results

### 3.1 Effects of Aquamin^®^ alone, the LPS-cytokine mix and the combination of the two interventions on proteomic signature: overview

Colon organoids from three subjects (grown individually and treated separately) were incubated for a 2-week period with subculture at the end of week-one under control conditions (i.e., in L-WRN–KGM Gold culture medium) or in the same medium supplemented with Aquamin^®^ at a concentration providing 3 mM calcium, with the LPS-cytokine mix (1:250 dilution of stock solution) or with a combination of both interventions. At the end of the 2-week incubation period, protein was prepared from each of the treatment groups (*n* = 3 subjects kept separately) and evaluated by proteomic analysis. From these separate evaluations, a total of 4,700 unique proteins was identified with <2% FDR. Using a 1.8-fold difference from control and <2% FDR as criteria, these studies identified a total of 869 proteins altered with Aquamin^®^ alone, 219 proteins altered with the LPS-cytokine mix and 457 proteins altered with the combination of the two interventions. Not surprisingly, the two interventions (Aquamin^®^ alone and LPS-cytokines) were very different in the proteins affected and there was essentially no concordance and little overlap between the two interventions (Supplementary Figure S2A). In contrast, when proteins altered in response to the LPS-cytokine mix with or without Aquamin^®^ were compared, there was considerable overlap (Supplementary Figure S2B). Likewise, when proteins altered in response to Aquamin^®^ treatment with or without the LPS-cytokine mix were compared, significant overlap was observed (Supplementary Figure S2C).

After evaluating each specimen separately, data from the three specimens were merged to obtain mean abundance ratio values. Using the same criteria (1.8-fold change from control with <2% FDR), a total of 91 proteins were altered in response to Aquamin^®^ (alone), while 94 proteins were altered in response to the pro-inflammatory mix and 145 proteins were altered with the combination of these two interventions (Supplementary Figure S3A). The majority of proteins were upregulated (independent of intervention) with only a small fraction of the total being downregulated. Venn plots show minimal overlap among the three treatment groups (Supplementary Figure S3B). The number of unique proteins was greater with the multi-mineral intervention alone or in concomitant treatment with LPS-cytokines than with the LPS-cytokine treatment alone. The heatmap (Supplementary Figure S3C) provides an overview of the differentially expressed proteins (proteomic profile) with a 1.8-fold change in response to each intervention and also shows corresponding data from each intervention for comparison.

As part of the comparison of proteomic signatures, protein abundance ratios from the three interventions were plotted using volcano plots. Supplementary Figure S4 shows the fold-change distribution of individual proteins responsive to each intervention using volcano plots. Significantly altered (at *p* < 0.05) upregulated proteins are shown in blue while downregulated proteins are shown in red. The bold blue and red colors indicate proteins that were statistically different and also met the criteria of at least 1.8-fold up- or downregulation (Supplementary Figure S4). These findings show overall differences or similarities between and/or among the signatures under the influence of the three interventions.

### 3.2 Human colon organoid epithelial differentiation and barrier protein expression: effects of Aquamin^®^ alone, the LPS-cytokine mix and the combination of the two interventions

**FIGURE 1:**
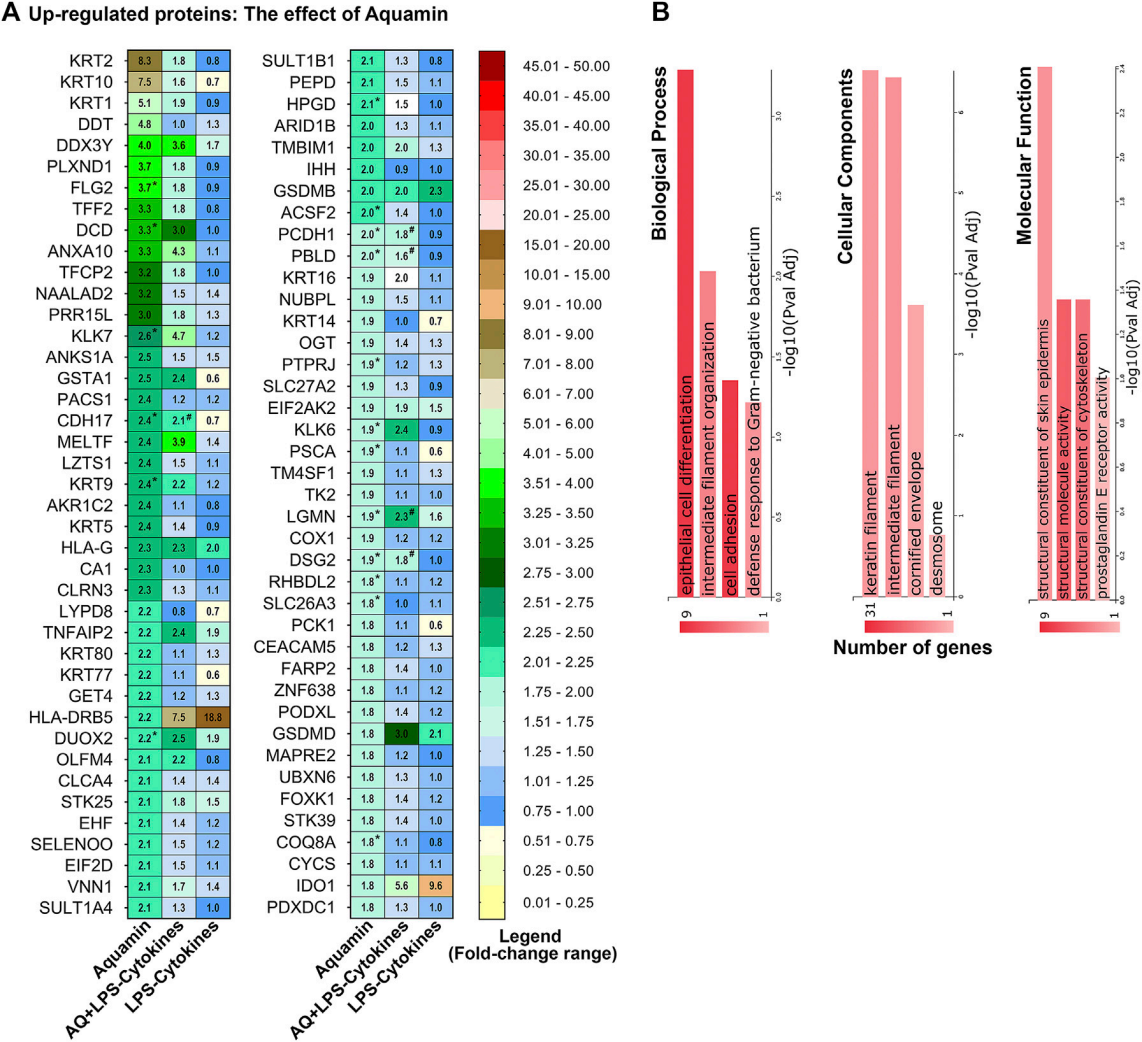
Upregulated proteins: The effect of Aquamin^®^ alone on the proteomic expression of human colon organoids. **(A)** Heatmap. Values represent average abundance ratio from organoids (*n* = 3 subjects) as compared to the control. Aquamin alone treatment (left column): These differentially expressed proteins were upregulated at 1.8-fold change (<1% FDR except NAALAD2 and TK2 which are <2%FDR) in response to Aquamin. Corresponding average abundance ratios are provided from the other two treatment groups for comparison. (*) Represents significance in upregulation as compared to the control and (#) represents significance as compared to LPS-cytokines (at *p* < 0.05). **(B)** Top biological processes, cellular components and molecular functions curated by GeneCodis functional enrichment for the proteins (upregulated by Aquamin^®^) shown in Figure 1A.

Figure 1A displays a complete list of 81 proteins upregulated in response to Aquamin^®^—i.e., the multi-mineral intervention—based on an unbiased approach with a cutoff of 1.8-fold. Individual proteins (listed in Figure 1A) are identified in Supplementary Table S3. Consistent with what we have reported previously in colon organoids derived from tissue of healthy individuals (Attili et al., 2019) or from UC lesional tissue (Aslam et al., 2020a), Aquamin^®^ alone caused upregulation of several keratins and other differentiation-related proteins. Two signature proteins related to epithelial cell cohesion and tissue strength (i.e., desmoglein-2 and cadherin-17) were strongly upregulated by the multi-mineral product as was trefoil factor-2 (a component of the mucinous layer) and carcinoembryonic antigen related cell adhesion molecule-5 (CEACAM-5). Figure 1B shows some of the top biological processes, cellular components and molecular functions influenced by these Aquamin^®^-sensitive proteins. These involve epithelial cell differentiation, cell adhesion, and structural and cytoskeletal molecule activity. Cellular components involve keratin and intermediate filaments and desmosomes. These functional enrichment data strengthen the notion that Aquamin^®^ enhances these components while improving barrier structure. Supplementary Table S4 provides a complete list of the functional enrichment components including Reactome, KEGG and Panther pathways affected by the proteins upregulated with Aquamin^®^ at 1.8-fold-change. Formation of the cornified envelope, keratinization, type I hemidesmosome assembly, cell junction organization and cadherin signaling pathway were among the significantly upregulated pathways (Supplementary Table S4).

**FIGURE 2:**
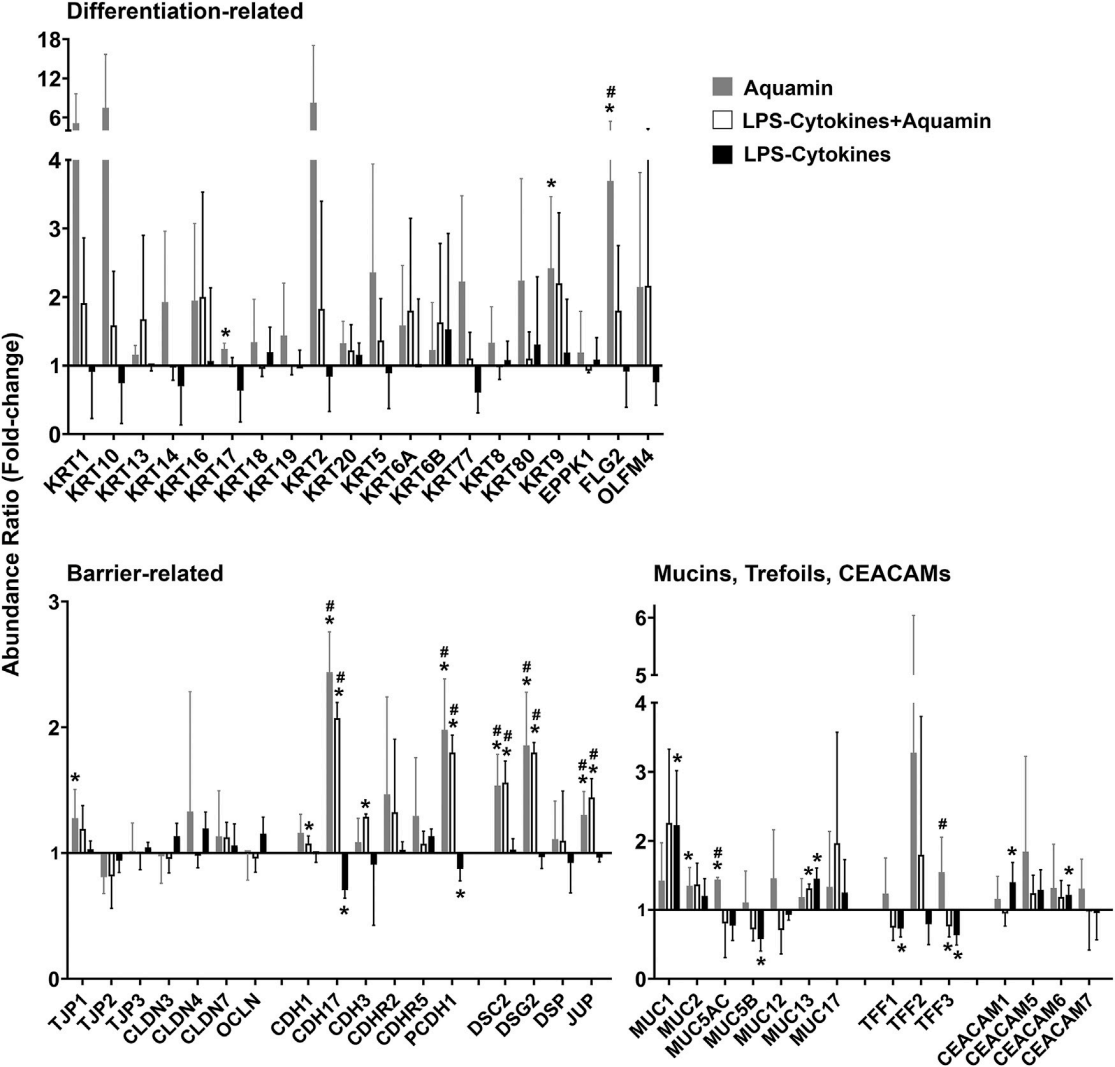
Directed search for proteins involved in differentiation and barrier formation: Effects of Aquamin^®^ alone compared to Aquamin^®^ in the presence of the LPS-cytokine mix. Bar graphs showing the up- or downregulation of individual proteins of interest (merged data; *n* = 3 subjects; 3 separate proteomic runs) with each of three interventions compared to control (independent of fold-change but with <2% FDR). Values shown are means and standard deviations. (*) represents significance (*p* < 0.05) relative to the control and (^#^) represents significance (*p* < 0.05) relative to the corresponding LPS-cytokine mix.

To obtain a more complete picture of how Aquamin^®^ altered differentiation and barrier protein expression, the proteomic database was searched specifically for proteins involved in differentiation, tissue strength and barrier function. Proteins of relevance are presented in Figure 2. In addition to the proteins detected in the nonbiased search (Figure 1 and Supplementary Table S3 above), several additional keratins and other differentiation-related proteins were identified along with a number of adherens junctional proteins (cadherin family members), desmosomal proteins, tight junctional proteins, trefoils, mucins, and CEACAM components. Consistent with our past reports (Attili et al., 2019; Aslam et al., 2020a), multiple cadherins and desmosomal proteins demonstrated strong upregulation with Aquamin^®^. Also consistent, most of the tight junctional proteins changed little in response to Aquamin^®^, although claudin-4 and ZO1 (TJP1) were upregulated.

Effects of the LPS-cytokine mix on the same Aquamin^®^-responsive proteins shown in Figures 1, 2 are presented for comparison in both figures. As seen, virtually none of the differentiation and barrier proteins were upregulated in response to the LPS-cytokines treatment alone. Several, in fact, demonstrated decreased expression, consistent with the barrier-disrupting effects of inflammation (Bruewer et al., 2003; Bandyopadhyay et al., 2021). Most importantly, Figures 1, 2 show that even in the presence of the pro-inflammatory stimulus, the multi-mineral intervention was able to stimulate significant upregulation of proteins critical to differentiation and barrier formation. Among these were cadherin-17, protocadherin-1, desmocollin-2, desmoglein-2, and junction plakoglobin. The only exception was muc1, a protein known to be strongly expressed in the inflamed mucosa as part of the host response (Li et al., 2003). Supplementary Table S5 highlights the pathways and functional enrichment elements (biological processes, cellular components, and molecular functions) by the proteins presented in Figure 2. Some top-listed enrichment features were epithelial cell differentiation, maintenance of gastrointestinal epithelium, protein localization to cell-cell junctions, cadherin binding, scaffold protein and calcium ion binding. The main cellular components involved with these altered proteins were cornified envelope, cell junction, cell-cell junction and desmosome. The affected pathways with proteins modified with both Aquamin^®^ groups (Aquamin^®^ in the absence or presence of LPS-cytokines) were cell junction organization, cell-cell communication, cell-cell junction organization, adherens junctions interactions, cadherin signaling pathway and Wnt signaling pathway.

### 3.3 Barrier protein expression assessment in human colon organoids by confocal immunofluorescence: effects of Aquamin^®^ alone, the LPS-cytokine mix and the combination of the two interventions

**FIGURE 3:**
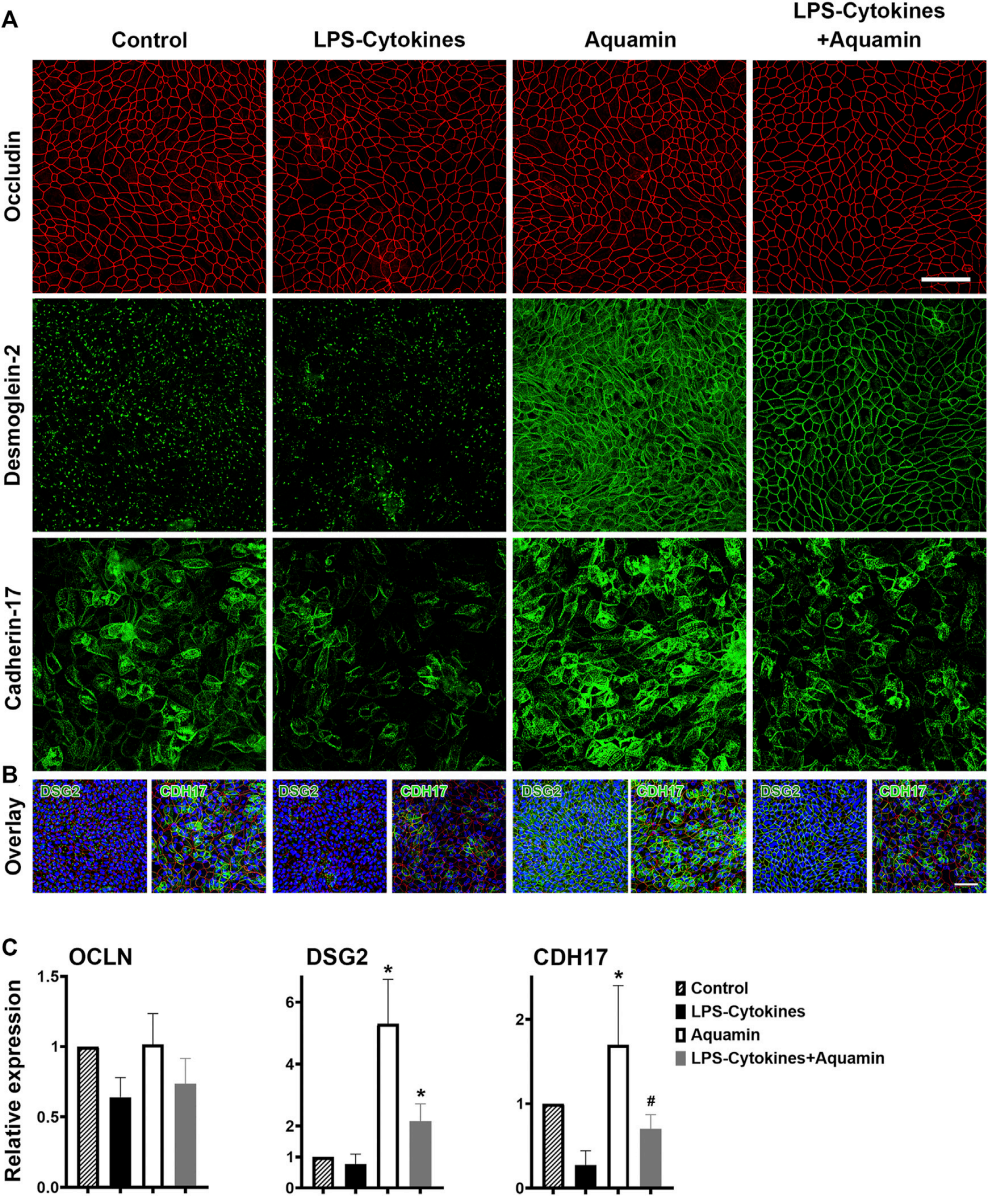
Confocal fluorescent microscopic images of occludin, desmoglein-2 and cadherin-17. **(A)** Colon organoids were plated on transwell membranes and incubated under the indicated conditions. After the TEER assessment on day-3, membranes were prepared and stained. Membranes were stained with a combination of antibodies to occludin (red) or desmoglein-2 (green) or a combination of occludin (red) and cadherin-17 (green). Upper panels: Occludin (max projected); Middle panels: Desmoglein-2 (max projected); Lower panels: Cadherin-17 (max projected). For confocal microscopic imaging, 54, 49, 47, and 36 z-planes were captured for desmoglein-2-stained images, while for cadherin-17 staining 26, 39, 32, and 38 z-planes captured for control, LPS-cytokines, Aquamin^®^ and LPS-cytokines supplemented with Aquamin^®^ respectively. Scale bar = 60 µm. **(B)** Overlay expression for desmoglein-2 (green) or cadherin-17 (green), occludin (red) and DAPI (blue) is shown for each condition (each panel shown in Figure 3A). The nuclear (DAPI) staining clearly demonstrates a full cell layer in each of the four conditions. Scale bar = 60 µm. **(C)** Quantitative confocal expression. ImageJ was employed to quantify the intensity of the confocal microscopy-generated images (z-stacks) for all three stains (occludin, desmoglein-2 and cadherin-17) from 2 separate experiments. For each staining expression, the control expression was used for the comparison. (*) indicates statistical significance from control and LPS-cytokines, while (^#^) indicates a statistical difference from the LPS-cytokine mix (*p* < 0.05; calculated by two-tailed unpaired *t*-test). Supplementary Figure S5 presents additional views of occludin and desmoglein-2-stained confocal fluorescent microscopic images at 45°–50° angle from 3D renderings.

Given the strong and significant upregulation of both desmoglein-2 and cadherin-17 by Aquamin^®^ in the proteomic assessment with and without LPS-cytokine treatment and downregulation by the LPS-cytokine mix alone (Figures 1, 2 and Supplementary Figure S4), the two proteins were assessed by confocal fluorescence microscopy. Occludin was evaluated in parallel with both proteins. Confocal staining results are presented in Figure 3A (occludin in red; desmoglein-2 in green; cadherin-17 in green). Under control conditions, desmoglein-2 was readily detected as punctate (green) fluorescence throughout the section, but no distinct cell surface expression was seen. Specifically, no distinct cell-cell boundaries were seen in desmoglein-2-stained sections under control conditions. The punctate appearance with desmoglein-2 staining in the control is consistent with our past findings (McClintock et al., 2020) and the findings of others (Chitaev and Troyanovsky, 1997; Cirillo et al., 2008) and strongly supports the conclusion (Chitaev and Troyanovsky, 1997; Cirillo et al., 2008) that in the absence of sufficient calcium to maintain cell-cell adhesion, this protein will be mostly cytoplasmic. The cytoplasmic versus surface staining pattern was confirmed using a rotating 3D animation of confocal z stacks (Supplementary Movie S1) and snapshots taken from the movie files at 45–50-degree angles (Supplementary Figure S5). A similar pattern of fluorescence was observed in the section from the LPS-cytokine-treated organoid culture. In contrast, Aquamin^®^ treatment dramatically increased detection of desmoglein-2. Cell-cell boundaries were clearly articulated between adjacent cells. The strong surface staining pattern in Aquamin^®^-treated cultures was confirmed using a rotating 3D animation of confocal z stacks (Supplementary Movie S1 and Supplementary Figure S5). Consistent with proteomic findings, treatment with the mix of LPS and cytokines had little adverse effect on desmoglein-2 staining in the presence of Aquamin^®^.

With cadherin-17 (Figure 3A), cell borders could be identified under all four treatment conditions based on the pattern of green fluorescence observed. Differences in staining intensities distinguished groups. The most intense staining was seen in sections from Aquamin^®^-treated organoids while the weakest was observed in response to the LPS-cytokine mix in the absence of Aquamin^®^. The combination of Aquamin^®^ and the LPS-cytokine mix produced an intermediate staining intensity.

Also consistent with the proteomic findings presented here and with previous confocal assessment (McClintock et al., 2020), neither intervention modulated occludin expression significantly. Distinct cell boundaries (red fluorescence) were consistently seen under all conditions (Figure 3A). Variations in intensity were slight. Figure 3B provides an overlay with DAPI for each panel shown in Figure 3A. The nuclear stain clearly demonstrates a full cell layer in each of the four conditions. Finally, a quantitative assessment of the staining intensity by ImageJ is presented in Figure 3C. For desmoglein-2 staining, both Aquamin^®^ groups have significantly increased expression compared to both control and LPS-cytokine conditions. Regarding the expression of cadherin-17, Aquamin^®^ alone was significantly increased compared to control and LPS-cytokines while Aquamin^®^ in the presence of LPS-cytokines was only significant compared to the pro-inflammatory mix. The intensity of occludin staining was not different among the groups except for the modest increase with Aquamin^®^ (Figure 3C).

### 3.4 Human colon organoid structure and barrier properties: effects of Aquamin^®^ alone, the LPS-cytokine mix and the combination of the two interventions

**FIGURE 4:**
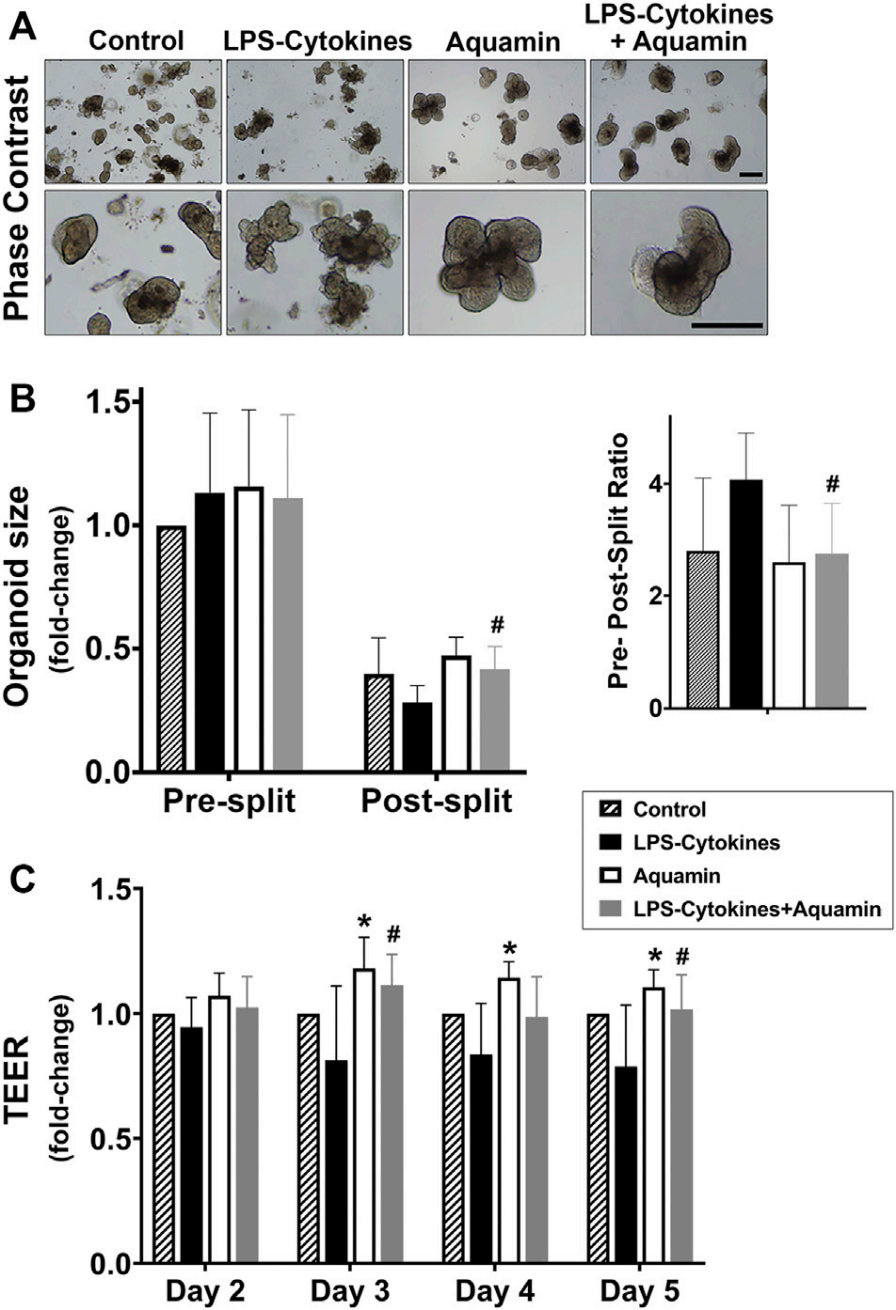
Colon organoid appearance, tissue cohesion and transepithelial electrical resistance (TEER) assessment. **(A)** At the end of the incubation period, intact colon organoids were examined by phase-contrast microscopy. Organoids were present as thick-walled structures with few surface buds. A wide range of sizes and shapes were seen under all conditions. Images in the upper panels represent organoids at lesser magnification and the lower panels represent at higher magnification. Scale bars = 500 μm. **(B)** Colon organoids were maintained in culture under the designated conditions. At the end of the incubation period, the size of multiple individual organoids was assessed by measuring surface area in phase-contrast images. Following this, the colon organoids were harvested and fragmented for subculture. After plating, the fragmented organoids were measured again. On the left, mean values of size (and standard deviations) are presented of individual organoids from 10–12 power fields at 40X with a total of 100 organoids per condition in both pre- and post-split cultures. On the right, a pre-, post-split mean ratio is shown representing the change in surface area in the organoids from the respective groups. Data were compared for statistical differences using ANOVA followed by unpaired-group comparisons. (^#^) indicates a statistical difference from the LPS-cytokine mix only at *p* < 0.05. **(C)** Colon organoids were seeded on transwell membranes to obtain a 2-D monolayer and incubated under the designated conditions. On days 2, 3, 4, and 5, transepithelial electrical resistance across the cell layer was assessed. Values shown are mean fold-change (and standard deviations) as compared to the control conditions. This assay is based on four (for Day 2 and Day 3) and three (for Day 4 and Day 5) separate experiments with 2–3 membranes per condition per experiment. Pooled TEER data were analyzed for statistical differences using ANOVA followed by unpaired-group comparisons. (*) indicates statistical significance from control, while (^#^) indicates a statistical difference from the LPS-cytokine mix (*p* < 0.05; calculated by two-tailed unpaired *t*-test).

Colon organoids from the three subjects were incubated individually for a 2-week period (with subculture at the end of week-one) under the same conditions as used above (i.e., control conditions or in medium supplemented with Aquamin^®^, with the LPS-cytokine mix or with a combination of both interventions. At the beginning of the treatment phase, organoids were approximately 40 µm in size (on average). At the time of harvest (either after the first week or at the end of the 14-day culture period), individual organoids had increased to approximately 500 µm in diameter in all treatment groups. Phase-contrast images taken at the end of the 2-week incubation period identified no gross morphological features attributable to either intervention alone or with the combination of interventions compared to organoids grown in the control medium. Individual organoids appeared as thick-walled, round or oval-shaped, structures. Some of the structures were multi-lobed (Figure 4A).

As part of the analysis, the colon organoids were assessed for cell-cell cohesion and the results are presented in Figure 4B. For these studies, surface area measurements were made on multiple individual organoids (i.e., a minimum of at least 100 individual organoids in each treatment group—10 to 12 40X power fields per condition as described in Section 2). Following this, subculture was performed, and individual organoids were “sized” again in the same manner and compared to the pre-split organoids. With organoids maintained under control conditions, there was an approximately 2.5-fold reduction in organoid size during subculture (i.e., average post-split size of individual organoids compared to average pre-split size). With organoids incubated in a culture medium containing the LPS-cytokine mix in the absence of Aquamin^®^, a 4.1-fold size reduction was seen (i.e., the organoids had fragmented into smaller pieces). With the multi-mineral intervention alone, pre-split versus post-split differences in organoid size were slightly reduced as compared to the control (i.e., 2.4-fold size reduction). Finally, and most importantly, Figure 4B demonstrates that the capacity of Aquamin^®^ to maintain organoid cohesion was not compromised by the simultaneous treatment with the LPS-cytokine mix. Pre-split versus post-split differences in organoid size observed in the multi-mineral-treated organoids whether or not the organoids were concomitantly exposed to the pro-inflammatory stimulus were similar (Figure 4B).

In parallel studies, colon organoids were plated on collagen IV-coated transwell membranes as described in the Materials and Methods Section. One day later, replicate transwell membranes were incubated with control medium alone or exposed to Aquamin^®^, the LPS-cytokine mix or a combination of the two. Electrical resistance across the cell layer was used as a functional barrier assay. Substantial electrical resistance across the cell layer was seen under all experimental conditions beyond day-2. For example, under control (day-2) conditions, an average TEER value of 567 ± 217 Ω per cm^2^ (*n* = 4 separate experiments) was seen. By day-4 and day-5, this value had risen to 984 ± 279 and 900 ± 202 Ω per cm^2^. As seen in Figure 4C, compared to control conditions, Aquamin^®^ alone increased TEER values on days 3, 4 and 5 and these differences were significant as compared to control condition. In contrast, LPS-cytokine treatment significantly reduced electrical resistance by 19% and 21.2% of control on day 3 and day 5 respectively (*p* < 0.05), consistent with barrier-disrupting effects in an inflammatory environment. Most importantly, the average TEER values obtained in transwell membranes exposed to the combination of interventions (Aquamin^®^ in the presence of LPS-cytokines) were 11.2% and 2% above the control and increased 36% and 29% compared to LPS-cytokine treatment alone on day 3 and day 5. These were significantly different from the LPS-cytokine values on day 3 and day 5 (Figure 4C).

### 3.5 Effects of the LPS-cytokine mix on proteomic signature: alone and in combination with Aquamin^®^

The data presented above demonstrate the capacity of the multi-mineral intervention to improve barrier structure and function in human colon organoids and, more importantly, to maintain barrier function in the presence of a strong pro-inflammatory stimulus. We next asked if, and to what extent, multi-mineral intervention could suppress other pro-inflammatory changes (i.e., independent of barrier) brought about by the LPS-cytokine mix. To do this, we searched the proteome database for proteins up- or downregulated in organoids exposed to the LPS-cytokine mix alone and then (for comparison) determined how the expression levels of the same proteins changed when Aquamin^®^ was included along with the LPS-cytokine mix.

**FIGURE 5:**
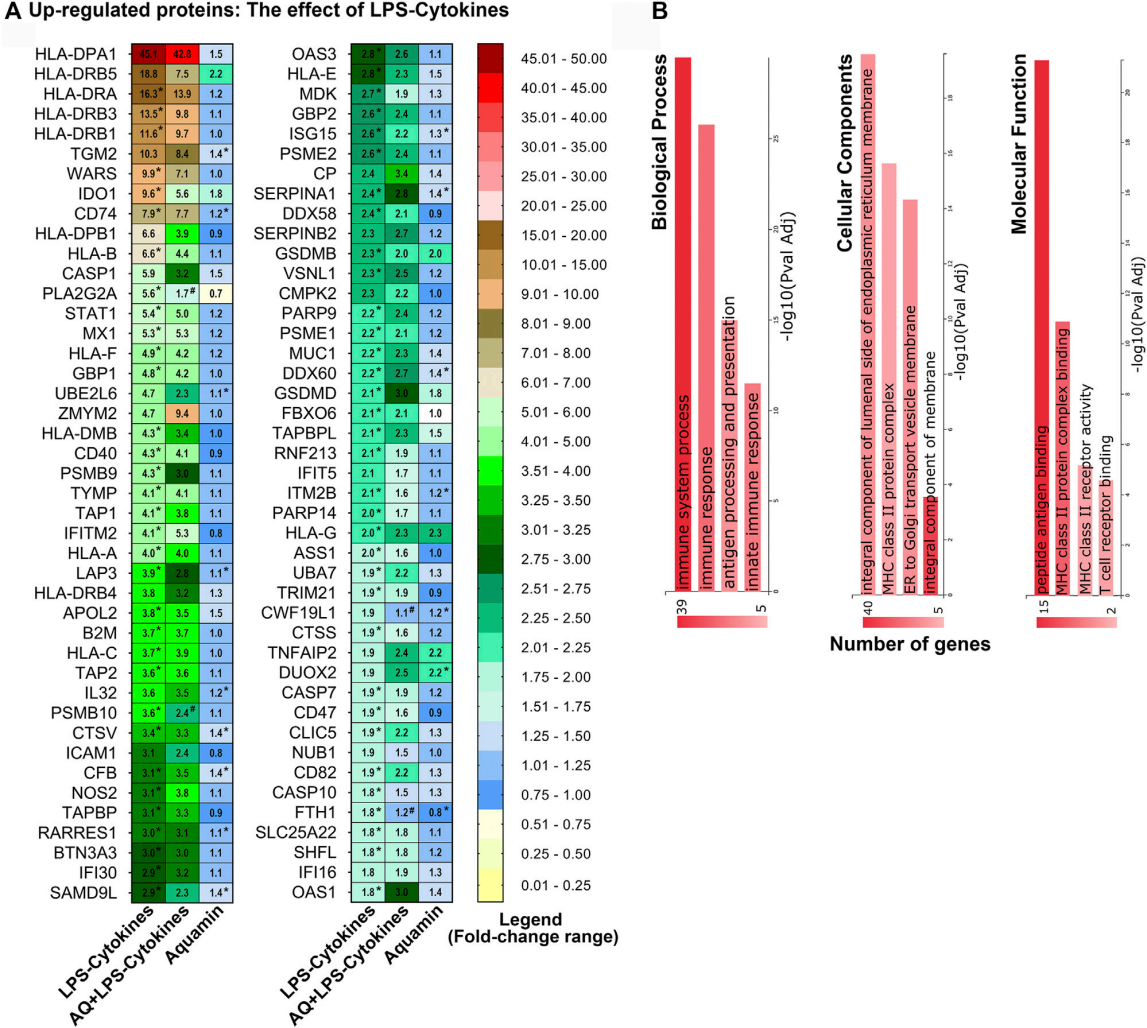
Upregulated proteins: The effect of LPS-cytokines on the proteomic expression of human colon organoids. **(A)** Heatmap. Values represent average abundance ratio from organoids (*n* = 3 subjects) as compared to the control. LPS-cytokines alone treatment (left column): These differentially expressed proteins were upregulated at 1.8-fold change (<1% FDR) in response to the pro-inflammatory mix. Corresponding average abundance ratios are provided from the other two treatment groups for comparison. (*) Represents significantly altered proteins as compared to the control and (#) represents significance as compared to LPS-cytokines (at *p* < 0.05). **(B)** Top biological processes, cellular components and molecular functions curated by GeneCodis functional enrichment for the proteins altered by LPS-cytokines as shown in Figure 5A.

Figure 5A (and Supplementary Table S6) provide a list of the 86 individual proteins upregulated in response to the LPS-cytokines stimulus with at least a 1.8-fold change from control. Table 1 shows the eight downregulated moieties affected by LPS-cytokine treatment alone; Figure 5B (and Supplementary Table S7) shows functional enrichment data related to the proteins upregulated by the LPS-cytokine stimulus. Additional pathways affected by protein changes resulting from the pro-inflammatory stimulus are presented in Supplementary Tables S7, S8 (up- or downregulated respectively). Among the most highly upregulated proteins were several HLA class II histocompatibility antigens along with certain HLA class I antigens. Additional proteins that support antigen presentation, inflammation, cytokine signaling, PD-1 signaling and immune cell recognition were induced along with HLA moieties. Not surprisingly, upregulation of immune cell signaling pathways accompanied these changes. The cell structures associated with these proteins are integral components of cellular membrane and part of the MHC class II protein complex. Peptide antigen binding, MHC class II protein complex binding/receptor activity and T cell receptor binding are among the top molecular functions influenced by these proteins (Figure 5 and Supplementary Table S7).

**TABLE 1:** Downregulated proteins: The effect of LPS-cytokines on the proteomic expression.

Proteins	Genes	Treatment groups		
		LPS-cytokines	LPS-cytokines + Aquamin	Aquamin
Proteasome subunit beta type-6	PSMB6	**0.31 ± 0.28** ^a^	0.36 ± 0.20^a^	1.03 ± 0.22^b^
Proteasome subunit beta type-5	PSMB5	**0.52 ± 0.40**	0.70 ± 0.35	1.01 ± 0.03^b^
Proteasome subunit beta type-7	PSMB7	**0.53 ± 0.25** ^a^	0.54 ± 0.20^a^	0.99 ± 0.18^b^
Antileukoproteinase	SLPI	**0.55 ± 0.37** ^a^	0.60 ± 0.32^a^	0.91 ± 0.27
Regulator of G-protein signaling 16	RGS16	**0.55 ± 0.28** ^a^	0.60 ± 0.27^a^	1.48 ± 0.88
Elafin	PI3	**0.55 ± 0.07** ^a^	0.46 ± 0.02^a^	1.17 ± 0.34^b^
Phosphoenolpyruvate carboxykinase, cytosolic [GTP]	PCK1	**0.55 ± 0.24** ^a^	1.10 ± 0.42	1.84 ± 1.02^b^
Glutathione S-transferase A1	GSTA1	**0.56 ± 0.24** ^a^	2.42 ± 3.12	2.45 ± 1.87

Values represent average abundance ratio from organoids (*n* = 3 subjects) as compared to the control ±standard deviation. LPS-cytokine treatment (bold): These proteins were downregulated at 1.8-fold change (<1% FDR) in response to LPS-cytokine mix. Corresponding average abundance ratios are provided from the other two treatment groups for comparison.

^a^ Represents significance as compared to the control.

^b^ Represents significance as compared to LPS-cytokines (at *p* < 0.05).

Several proteins involved in intracellular protein processing/breakdown (degradative enzymes and oxidant generators) were also responsive to the LPS-cytokine mix, as were several moieties involved in ubiquitinating reactions and multiple proteasome subunits (Figure 5 and Supplementary Table S6). Of interest, the most highly upregulated proteasome subunits (PSMB9, PSMB10, PSME2, and PSME1) are found in the specialized proteasome complex known as the immunoproteasome (Nathan et al., 2013), which is responsible for the modification of peptides for binding by major histocompatibility complex molecules and presentation to T cells. These proteins (PSMB10, PSME1, PSME2, and PSMB9) are also involved in ornithine decarboxylase upregulation. Increased colonic ornithine decarboxylase activity occurs with mucosal inflammation (Allgayer et al., 2007) and is thought to play a role in the pathogenesis of colitis. As noted above, muc-1, a cell surface mucin, was upregulated in response to LPS-cytokines. It has been shown previously that pro-inflammatory cytokines increase muc1 expression in the oral mucosa as part of the host response (Li et al., 2003).

Among the proteins most downregulated in response to the LPS-cytokine mix (Table 1) were three proteasome subunits; all three of these proteins are present in the “classical” proteasome complex (Nathan et al., 2013); their downregulation is consistent with their replacement by analogues found in the immunoproteasome (Table 1). Another downregulated protein shown in Table 1 was glutathione S-transferase A1 (GSTA1). This protein is part of the glutathione antioxidant mechanism and a critical component of detoxification pathways (Langmann et al., 2004).

Table 1 and Figure 5 along with Supplementary Table S6 also show how the concomitant presence of Aquamin^®^ along with the LPS-cytokine mix affected the same proteins. As seen from Supplementary Table S6 and Figure 5, the majority of proteins that were upregulated in response to the LPS-cytokine mix alone also responded to the pro-inflammatory challenge when Aquamin^®^ was concomitantly present. This suggests that Aquamin^®^ does not act widely to suppress protein changes that occur in response to LPS-cytokine stimulation. In further support for this idea, Reactome pathway analysis conducted using proteins upregulated by the LPS-cytokine mix in the presence of Aquamin^®^ demonstrated a similar spectrum of target pathways affected as seen with the LPS-cytokine mix alone (Supplementary Table S9).

Although the overall effect of Aquamin^®^ on organoid response to LPS-cytokine stimulation was not widespread, there were several important exceptions. Specifically, among the 86 LPS-cytokine upregulated proteins, twenty-two were inhibited by at least 20% or more when Aquamin^®^ was concomitantly present. Among these were proteins known to play roles in modifying the pro-inflammatory environment as noted in Figure 5 and Supplementary Table S6. Included are Phospholipase A2, membrane associated (PLA2G2A), HLA class II histocompatibility antigen, DR beta 5 chain (HLA-DRB5), HLA class II histocompatibility antigen, DP beta 1 chain (HLA-DPB1) and Tryptophan-tRNA ligase, cytoplasmic (WARS). Of the eight proteins downregulated in organoids exposed to the LPS-cytokine mix (Table 1), the concomitant presence of Aquamin^®^ prevented reduced expression of two moieties (including, Glutathione S-transferase A1; a critical component of the cellular anti-oxidant shield).

In addition to proteins upregulated in response to Aquamin^®^, 10 downregulated proteins (nonbiased search; ≥1.8-fold with <2% FDR) were also identified (Supplementary Table S10A). Effects of LPS-cytokine treatment (alone and in the presence of Aquamin^®^) are presented in the same table for comparison. Of the ten proteins, five were significantly downregulated with Aquamin^®^ in the presence of a pro-inflammatory mix. Among the ten proteins were two of the three peptides that make up intact fibrinogen and the protein, SPARC. None of the three proteins was altered by the LPS-cytokine mix alone. Both fibrinogen—FGB and FGG (Davalos and Akassoglou, 2012; Zhang et al., 2021) and SPARC (Fonseca-Camarillo et al., 2021) have been shown to promote chronic inflammation, including in the gastrointestinal tract. Their downregulation by Aquamin^®^ suggests the possibility of an anti-inflammatory effect separate from barrier improvement. A list of pathways altered by the downregulated moieties with Aquamin^®^ is presented in Supplementary Table S10B.

In a final step, a directed search was carried out to identify additional Aquamin^®^-responsive proteins that may contribute to anti-inflammatory activity (including gut inflammation) through one mechanism or other (independent of cutoff value). Table 2 provides a list of such proteins. The proteins highlighted in the list were chosen based on 1) a potential role in inflammation (previously identified in the scientific literature), 2) a beneficial response to Aquamin^®^ (either up- or downregulation), and 3) failure of the LPS-cytokine mix to interfere with response to Aquamin^®^. Supplementary File S1 provides relevant citations to justify the inclusion of these proteins. Among proteins of interest identified in this manner is membrane-associated Phospholipase A2 (PLA2G2A). This Aquamin^®^-downregulated protein is a secreted form of the enzyme. In the gastrointestinal tract, it is upregulated in inflammation (Lambeau and Gelb, 2008), is responsible for epithelial cell damage and is thought to promote tumor formation (Escudero-Hernández et al., 2021). Also of interest, downregulation of fibrinogen chains by Aquamin^®^ could be expected to blunt abnormal toll-like receptor (TLR) signaling (Supplementary Table S10). Abnormal Toll-like receptor (TLR) signaling and dysfunctional TLR may contribute to the pathogenesis of inflammatory bowel disease (Cario, 2010).

**TABLE 2:** Proteins of interest (inflammation-related).

Proteins	Genes	Treatment groups		
		Aquamin	LPS-cytokines + Aquamin	LPS-cytokines
Fibrinogen beta chain	FGB	**0.43 ± 0.28** ^a^	**0.53 ± 0.27** ^a^	1.12 ± 0.70
Fibrinogen gamma chain	FGG	**0.46 ± 0.16** ^a^ ^,^ ^b^	**0.59 ± 0.24** ^a^	0.89 ± 0.20
SPARC	SPARC	**0.49 ± 0.06** ^a^ ^,^ ^b^	**0.54 ± 0.13** ^a^	0.87 ± 0.22
Phospholipase A2, membrane associated	PLA2G2A	**0.72 ± 0.25** ^b^	**1.74 ± 1.81** ^b^	5.60 ± 1.11^a^
Phosphoglycerate kinase 1	PGK1	**1.31 ± 0.40**	**1.33 ± 0.54**	0.99 ± 0.02
Protein kinase C alpha type	PRKCA	**1.31 ± 0.42**	**1.37 ± 0.39**	0.94 ± 0.11
Glutamine synthetase	GLUL	**1.40 ± 0.15** ^a^	**1.66 ± 0.67**	1.05 ± 0.34
Homeobox protein CDX-2	CDX2	**1.41 ± 0.21** ^a^ ^,^ ^b^	**1.35 ± 0.52**	0.97 ± 0.11
Fructose-1,6-bisphosphatase isozyme 2	FBP2	**1.47 ± 0.74**	**1.46 ± 0.85**	0.97 ± 0.09
Natural resistance-associated macrophage protein 2	SLC11A2	**1.50 ± 0.64**	**2.09 ± 1.21**	0.93 ± 0.10
NAD-dependent protein deacetylase sirtuin-3, mitochondrial	SIRT3	**1.64 ± 1.23**	**2.36 ± 2.36**	0.98 ± 0.24
Sialidase-1	NEU1	**1.66 ± 0.91**	**1.71 ± 0.93**	1.00 ± 0.26
Protein S100-A7	S100A7	**1.68 ± 0.23** ^a^ ^,^ ^b^	**2.26 ± 1.87**	0.95 ± 0.47
Phenazine biosynthesis-like domain-containing protein	PBLD	**1.95 ± 0.62** ^a^ ^,^ ^b^	**1.63 ± 0.29** ^a^ ^,^ ^b^	0.87 ± 0.08^a^
15-hydroxyprostaglandin dehydrogenase [NAD (+)]	HPGD	**2.05 ± 0.69** ^a^ ^,^ ^b^	**1.50 ± 0.61**	1.00 ± 0.16
ETS homologous factor	EHF	**2.12 ± 2.18**	**1.44 ± 0.66**	1.17 ± 0.28
Glutathione S-transferase A1	GSTA1	**2.45 ± 1.87**	**2.42 ± 3.12**	0.56 ± 0.24^a^
Dermcidin	DCD	**3.27 ± 1.41** ^a^ ^,^ ^b^	**3.05 ± 3.12**	1.00 ± 0.54

Values represent average abundance ratio from organoids (*n* = 3 subjects) as compared to the control ±standard deviation. Aquamin and Aquamin plus LPS-cytokine treatments (bold): These proteins were up- or downregulated regardless of the fold change (<1% FDR) in response to both Aquamin treatments. Corresponding average abundance ratios are provided from the LPS-cytokine treatment group for comparison.

^a^ Represents significance as compared to the control.

^b^ Represents significance as compared to LPS-cytokines (at *p* < 0.05).

## 4 Discussion

Our previous studies demonstrated that Aquamin^®^, the calcium-rich, magnesium-rich, multi-trace element-rich natural product used here, increased expression levels of several differentiation-related proteins and barrier proteins in human colon organoid culture (Attili et al., 2019; Aslam et al., 2020a; McClintock et al., 2020). Increased barrier protein expression was accompanied by improved barrier function (Schmitz et al., 1999; McGuckin et al., 2009; Salim and Söderholm, 2011; Baumgartner, 2013; Antoni et al., 2014; Ungewiß et al., 2017; Chang et al., 2018; Lee et al., 2018; Burkard et al., 2021). Findings in organoid culture were confirmed in a pilot-phase biomarker trial in healthy adults (Aslam et al., 2021). These past results highlight the importance of inorganic minerals to barrier formation in the gastrointestinal tract. They do not, however, indicate whether an effective barrier can be maintained in the face of an inflammatory attack. The findings presented here suggest that it can.

To summarize the present findings, organoid treatment over a 2-week period with a mix of LPS and three pro-inflammatory cytokines substantially reduced barrier function in organoids maintained under control conditions. In contrast, organoids provided with the multi-mineral supplement demonstrated improved barrier function under both control conditions and in the presence of the pro-inflammatory challenge. To the extent that barrier changes observed in colon organoid culture predict effects that will be seen in the human gastrointestinal tract, the use of the multi-mineral intervention would seem to make sense. Of course, demonstrating improvement *in vivo* will require controlled clinical studies. While such trials have not been completed at this point, a recent 90-day interventional trial with the same multi-mineral agent in healthy adults demonstrated a similar increase in barrier protein expression as observed in organoid culture (Aslam et al., 2021). Our ongoing 180-day trial (clinicaltrials.gov/: NCT03869905) is testing the same interventional agent for evidence of clinical benefit in individuals with UC as well as for direct effects on gut barrier function in both healthy adults and individuals with UC (clinicaltrials.gov/: NCT04855799). Results from the ongoing studies will go a long way toward establishing the potential for benefit with this approach in UC. Protein changes and permeability measurements will provide insight into therapeutic mechanisms. If warranted, the use of this GRAS-listed natural product could be included as an adjuvant treatment for UC, as to date there have been no safety or tolerability issues (Aslam et al., 2020b; Aslam et al., 2021). This low-cost, low-to-no toxicity product could provide the first of a kind intervention for barrier improvement in UC, mainly in subjects with mild to moderate disease.

Along with barrier enhancement, it is possible that a multi-mineral intervention could have additional activities that are directly anti-inflammatory. In one past study, Aquamin^®^ was reported to reduce symptoms of colitis in the 25-week IL-10−/− mouse model (Aviello et al., 2014). Our own 16-week study demonstrated reduced liver inflammation in NASH mice on a high-fat diet (Varani et al., 2022) while long-term (15–18 months) studies with healthy C57BL6 mice on a high-fat diet demonstrated reduced inflammation throughout the gastrointestinal tract, in the liver and systemically when Aquamin^®^ was incorporated into the diet (Aslam et al., 2010; Aslam et al., 2012). While all of these past murine studies point to a direct anti-inflammatory effect, the findings are also consistent with an improved colonic barrier being the primary target of the intervention with anti-inflammation being a consequent of the improved barrier. As part of the current study, therefore, the interventional agent was examined for direct effects on proteins known to drive inflammation in response to LPS-cytokine stimulation. As noted in the Results Section, the combination of LPS along with three potent pro-inflammatory cytokines strongly upregulated numerous proteins that participate in the inflammatory response. Not surprisingly, the various pathway analysis databases revealed the great extent to which these protein changes influenced the inflammatory landscape in the treated organoids.

While the overall response to the LPS-cytokine mix was not strongly impacted by Aquamin^®^ (in the presence of pro-inflammatory insult), the multi-mineral product did alter the expression of several individual proteins that have been linked to inflammation in the gastrointestinal tract in the same milieu. This included downregulation of proteins with known pro-inflammatory roles and upregulation of moieties with anti-inflammatory, antioxidant and anti-microbial functions. Whether Aquamin^®^’s effect on the expression of any of these individual proteins (alone or in combination) contributes in a meaningful way to a direct anti-inflammatory role is not known at this time and will have to await further work. Nonetheless, these findings suggest that in addition to barrier improvement, multi-mineral intervention could have other activities that directly counter inflammation in the gastrointestinal tract.

To be sure, there are limitations to the present work. Most importantly, although organoid culture technology provides a sophisticated model, no *ex vivo* approach can fully mimic the clinical situation. Acute flare-ups in UC are triggered by multiple factors. It is impossible to control for all these in any *in vitro* model. It should be noted, however, that the three cytokines provided here–i.e., TNF-α, IL-1β and IFN-γ–are all known to be present in the inflamed colonic mucosa (Hibiya et al., 2017; Jones et al., 2019; Nishimura et al., 2019; Pavlidis et al., 2022). Further, bacterial penetration of the normally impenetrable barrier provides a source of endotoxin (Johansson et al., 2014). Recent gene-expression profiling studies in inflammatory bowel disease have shown that multiple cytokines are, typically, responsible for the pattern of changes seen in most individuals (Pavlidis et al., 2022). It is not unreasonable to assume, therefore, that while organoid culture is not a perfect model, treatment of colonic organoids with a mix of pro-inflammatory stimuli can produce a milieu that approximates the inflamed human colonic mucosa. Therefore, the use of this technology to investigate potential interventions as carried out here with Aquamin^®^ or previously with other agents (Nishimura et al., 2019) would seem to be of value.

Finally, while UC is the focus of this work, this disease does not constitute the only clinical situation in which barrier defects are likely to be part of the pathophysiology. Barrier dysfunction has been suggested as a contributing factor in Crohn’s disease (Pearson et al., 1982) as well as in irritable bowel syndrome (Dunlop et al., 2006) and food allergies/malabsorption syndromes (e.g., celiac disease) (Pearson et al., 1982). Recent studies have also demonstrated the disruption of the colonic barrier in the setting of allogeneic hematopoietic cell transplantation (Tyszka et al., 2021), where barrier dysfunction and poor outcomes are linked. Perhaps most interestingly, high-fat intake as well as obesity, itself, are associated with compromised barrier function (Moreira et al., 2012; Gulhane et al., 2016). This provides a link to the systemic inflammatory changes seen in obesity and to the myriad of chronic inflammatory conditions associated with obesity. A low-cost, low-to no-toxicity approach to barrier maintenance could be of value in multiple clinical settings, but this will need to be established in future studies. The present work provides a rationale for these studies.

In summary, past studies have demonstrated that a multi-mineral intervention (Aquamin^®^) has the capacity to upregulate multiple proteins associated with barrier function in the gastrointestinal tract. The present studies suggest that even in the presence of a strong pro-inflammatory challenge, the beneficial effects on the colonic barrier protein expression are maintained. Further, even though the multi-mineral intervention did not affect the majority of proteins upregulated by the LPS-cytokine mix, several proteins that are part of the inflammatory process were, in fact, responsive to Aquamin^®^. Thus, a direct anti-inflammatory role for Aquamin^®^ appears to exist, along with (and independent of) the beneficial effects on barrier formation.

## Data Availability

The datasets presented in this study can be found as supplemental files or in online repository. The massspectrometry proteomics dataset is available on repository ProteomeXchange Consortium - PRIDE partner repository (https://www.ebi.ac.uk/pride/archive/projects/PXD038941) – dataset identifier PXD038941.
