# Supplementary material for: A multi-mineral intervention to counter pro-inflammatory activity and to improve the barrier in human colon organoids
Source: Front Cell Dev Biol. 2023 Jul 5;11:1132905. doi: 10.3389/fcell.2023.1132905 (PMC10354648; doi:10.3389/fcell.2023.1132905)
Supplement: Supplementary file 1 [file DataSheet1.zip › Supplementary Figure S1.PDF]

frontiers

Supplementary Material

A Multi-Mineral Intervention to Counter Pro-inflammatory Activity and to Improve the Barrier in Human Colon Organoids

James Varani[1], Shannon D McClintock[1], Daniyal M Nadeem[1], Isabelle Harber[1], Dania Zeidan[1], and Muhammad N Aslam[1*]

*** Correspondence:** Muhammad N Aslam; mnaslam@med.umich.edu

Supplementary Figure 1.

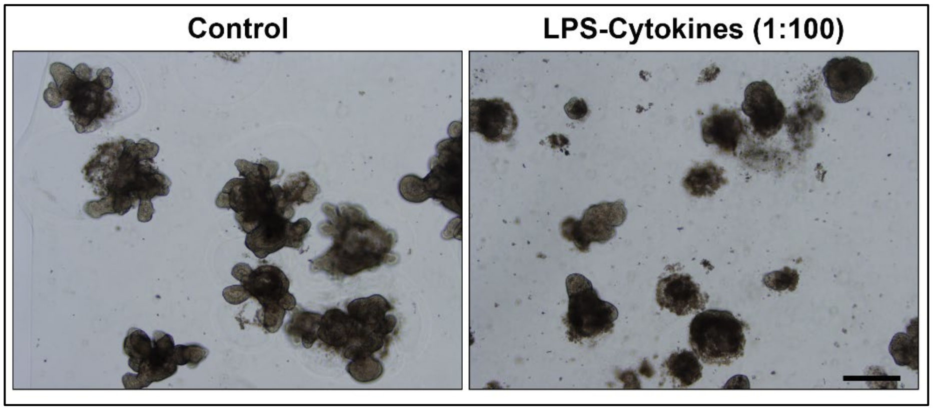

Supplementary Figure 1. Colon organoid appearance assessed by phase-contrast microscopy: Difference in appearance between control and LPS-cytokines-treated (at 1:100) organoids.

At the end of the incubation period, intact colon organoids were examined by phase-contrast microscopy. Control organoids (on left) were present as thick-walled structures with few surface buds. A wide range of sizes and shapes were seen under all conditions. While the organoids treated with higher concentrations of LPS-cytokines showed signs of toxicity. Toxicity was defined based on morphological appearance (evident by the presence of smaller organoids), and organoid failure to demonstrate features of differentiation (formation of thick walls and decreased budding structures). Scale bar=500µm.
