# Supplementary material for: A multi-mineral intervention to counter pro-inflammatory activity and to improve the barrier in human colon organoids
Source: Front Cell Dev Biol. 2023 Jul 5;11:1132905. doi: 10.3389/fcell.2023.1132905 (PMC10354648; doi:10.3389/fcell.2023.1132905)
Supplement: Supplementary file 1 [file DataSheet1.zip › Supplementary Figure S2.PDF]

frontiers

Supplementary Material

A Multi-Mineral Intervention to Counter Pro-inflammatory Activity and to Improve the Barrier in Human Colon Organoids

James Varani[1], Shannon D McClintock[1], Daniyal M Nadeem[1], Isabelle Harber[1], Dania Zeidan[1], and Muhammad N Aslam[1*]

*** Correspondence:** Muhammad N Aslam; mnaslam@med.umich.edu

Supplementary Figure 2.

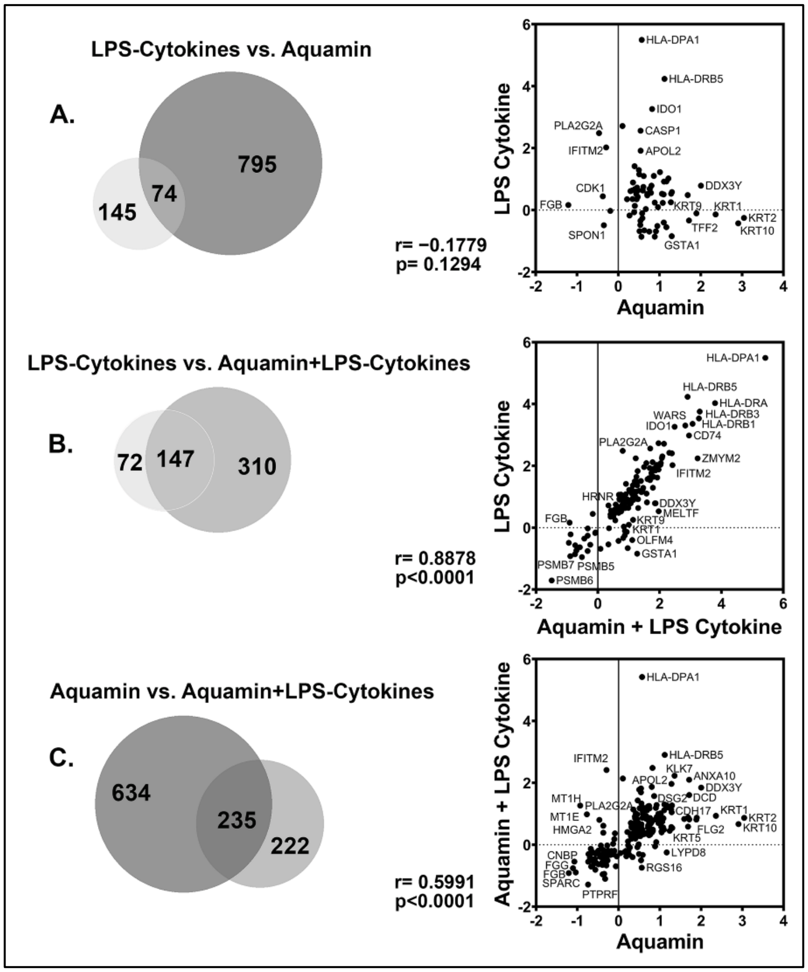

Supplementary Figure 2. Proteomic analysis of human colon organoids: Effects of the LPS-cytokine mix and Aquamin® alone and in combination – Correlation analysis.

At the end of the incubation period, lysates were subjected to TMT mass spectroscopy-based proteomic analysis. A (left): Venn plot showing proteins altered (increased or decreased) by an average of 1.8-fold or greater (with FDR<2%), comparing the LPS-cytokine mix with Aquamin®. The data are based on all unique proteins identified across organoid cultures from three separate specimens with each of the two interventions compared to the control. A (right): Correlation analysis showing relative expression levels of the seventy-four proteins (abundance ratio in log2 fold-change, 1.8-fold or greater) common to both interventions. B (left): Venn plot comparing proteins altered by the LPS-cytokine mix alone to those altered by the LPS-cytokine mix in the presence of Aquamin®. B (right): Correlation analysis showing relative expression levels of the 147 proteins common to both interventions. C (left): Venn plot comparing proteins altered in the presence of Aquamin® alone to the proteins altered by Aquamin® in combination with the LPS-cytokine mix. C (right): Correlation analysis showing relative expression levels of the 235 proteins common to both interventions.
