# Supplementary material for: A multi-mineral intervention to counter pro-inflammatory activity and to improve the barrier in human colon organoids
Source: Front Cell Dev Biol. 2023 Jul 5;11:1132905. doi: 10.3389/fcell.2023.1132905 (PMC10354648; doi:10.3389/fcell.2023.1132905)
Supplement: Supplementary file 1 [file DataSheet1.zip › Supplementary Figure S3.PDF]

Supplementary Material

A Multi-Mineral Intervention to Counter Pro-inflammatory Activity and to Improve the Barrier in Human Colon Organoids

James Varani[1], Shannon D McClintock[1], Daniyal M Nadeem[1], Isabelle Harber[1], Dania Zeidan[1], and Muhammad N Aslam[1*]

*** Correspondence:** Muhammad N Aslam; mnaslam@med.umich.edu

Supplementary Figure 3.

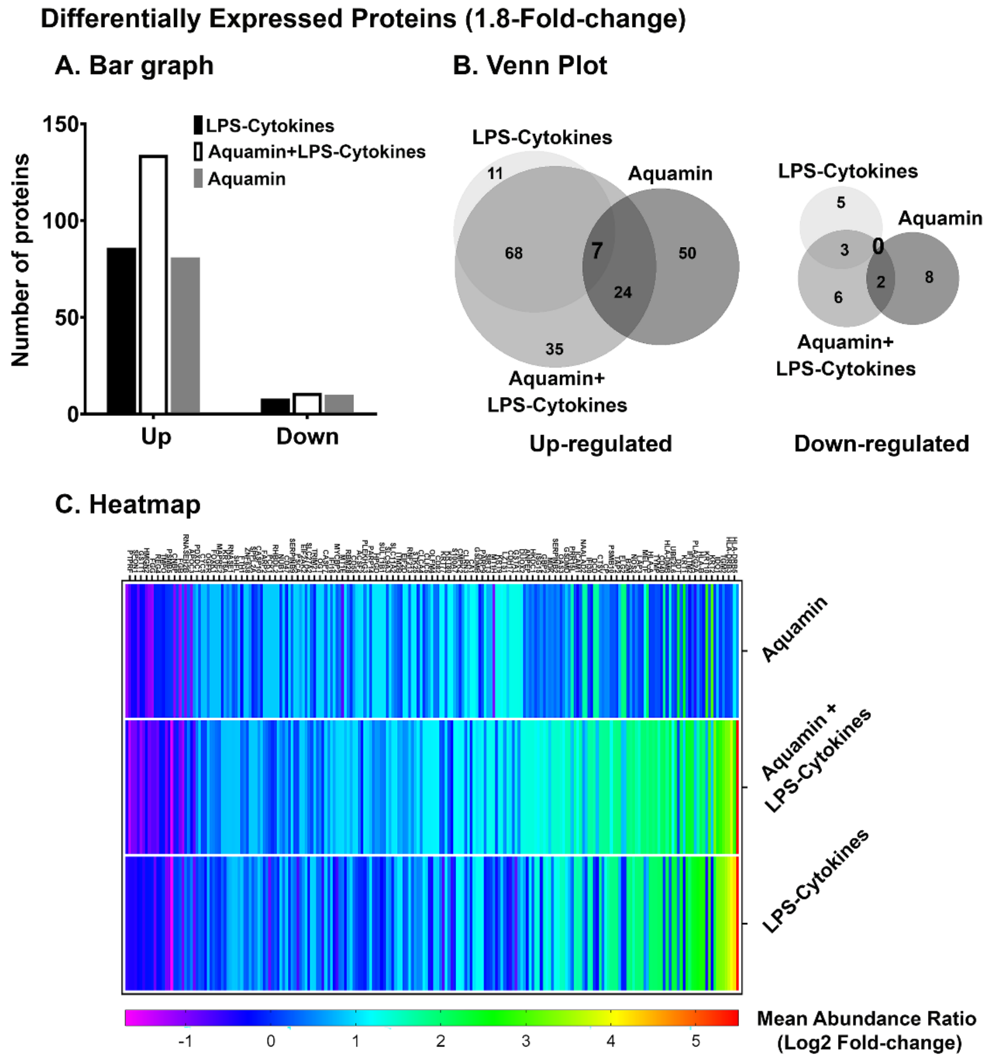

Supplementary Figure 3. Proteomic analysis of human colon organoids exposed to the LPS-cytokine mix, Aquamin® and the combination of both interventions: Data from three subjects merged. Proteomic data from the three separate colon organoid cultures from three subjects (analyzed in Supplement Figure 2) were merged to obtain mean values and re-analyzed. A: Bar graph showing proteins (merged data) increased or decreased by an average of 1.8-fold or greater with each of the three interventions compared to the control. B: Venn plots showing overlap among the three interventions. C: Heatmap displaying mean abundance ratio (log2 values) of differentially expressed proteins that are up- or down-regulated in response to each intervention across all three subjects.
