# Supplementary material for: A multi-mineral intervention to counter pro-inflammatory activity and to improve the barrier in human colon organoids
Source: Front Cell Dev Biol. 2023 Jul 5;11:1132905. doi: 10.3389/fcell.2023.1132905 (PMC10354648; doi:10.3389/fcell.2023.1132905)
Supplement: Supplementary file 1 [file DataSheet1.zip › Supplementary Figure S4.PDF]

frontiers

Supplementary Material

A Multi-Mineral Intervention to Counter Pro-inflammatory Activity and to Improve the Barrier in Human Colon Organoids

James Varani[1], Shannon D McClintock[1], Daniyal M Nadeem[1], Isabelle Harber[1], Dania Zeidan[1], and Muhammad N Aslam[1*]

*** Correspondence:** Muhammad N Aslam; mnaslam@med.umich.edu

Supplementary Figure 4.

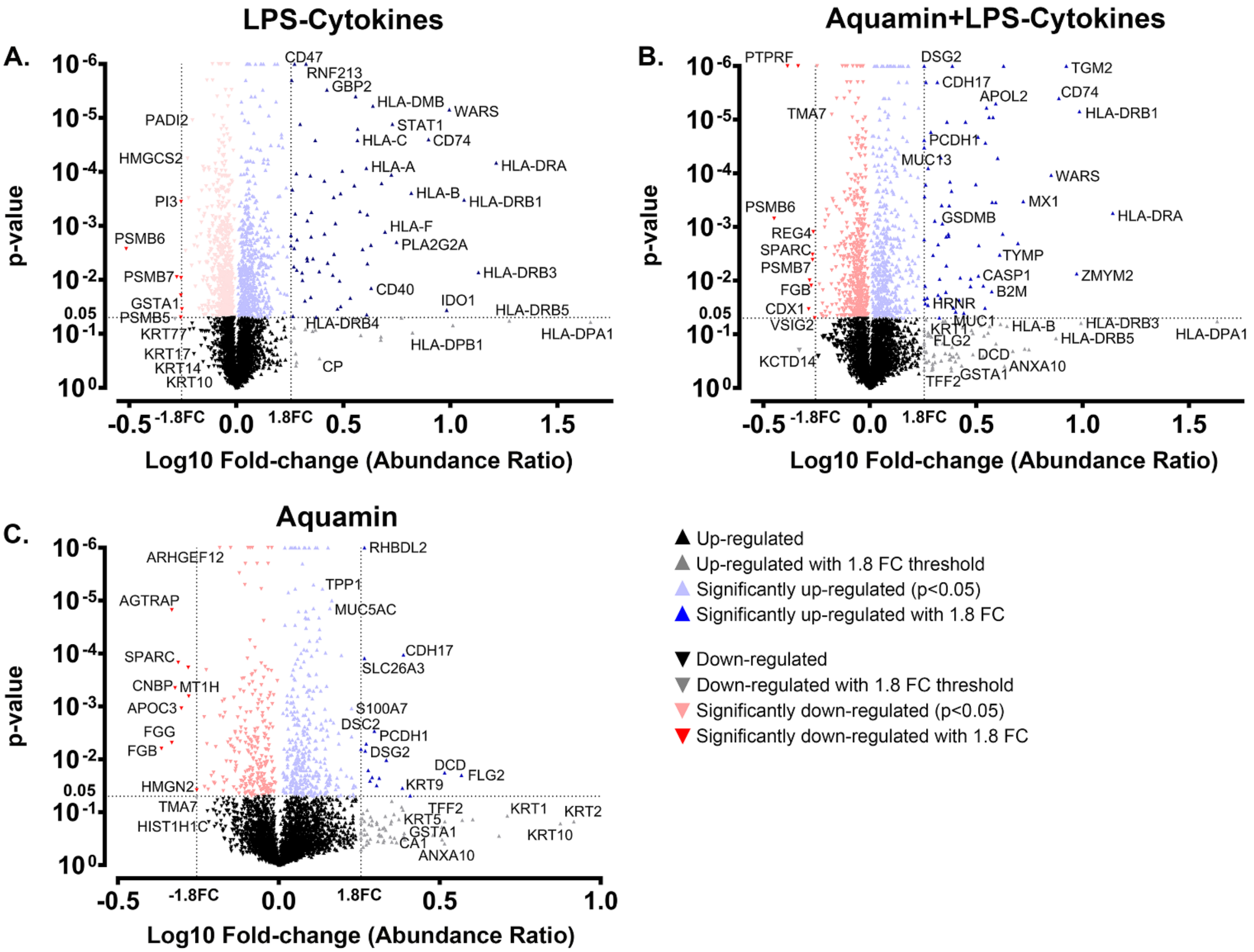

Supplementary Figure 4. Proteomic profile of human colon organoids in response to individual treatments. Volcano plots display the mean abundance of all 4700 proteins identified with <2% FDR. The distribution of up- or down-regulated proteins in response to each intervention compared to control. The x-axis shows the fold-change (log10) of individual proteins and the y-axis reflects individual protein p-values (n = 3 subjects per group). The proteins (not statistically significant) shown in black represent proteins that were <1.8-fold different from the control (up or down). Proteins shown with gray were different from the control by 1.8-fold or greater but not statistically different. Proteins shown in red represent down-regulated proteins and in blue represent up-regulated proteins that were statistically significant as compared to the control. Proteins in bold color (red or blue) represent statistically significant compared to the control and are different from the control by 1.8-fold change. A few individual proteins are labeled by protein (gene) name. Two dotted lines on the x-axis are representing 1.8-fold decrease or increase. One dotted line on the y-axis is representing significance ($p<0.05$). A: LPS-Cytokines. B: Aquamin® in the presence of LPS-Cytokines. C: Aquamin® alone.
