# Supplementary material for: A multi-mineral intervention to counter pro-inflammatory activity and to improve the barrier in human colon organoids
Source: Front Cell Dev Biol. 2023 Jul 5;11:1132905. doi: 10.3389/fcell.2023.1132905 (PMC10354648; doi:10.3389/fcell.2023.1132905)
Supplement: Supplementary file 1 [file DataSheet1.zip › Supplementary Figure S5.PDF]

Supplementary Material

A Multi-Mineral Intervention to Counter Pro-inflammatory Activity and to Improve the Barrier in Human Colon Organoids

James Varani[1], Shannon D McClintock[1], Daniyal M Nadeem[1], Isabelle Harber[1], Dania Zeidan[1], and Muhammad N Aslam[1*]

*** Correspondence:** Muhammad N Aslam; mnaslam@med.umich.edu

Supplementary Figure 5.

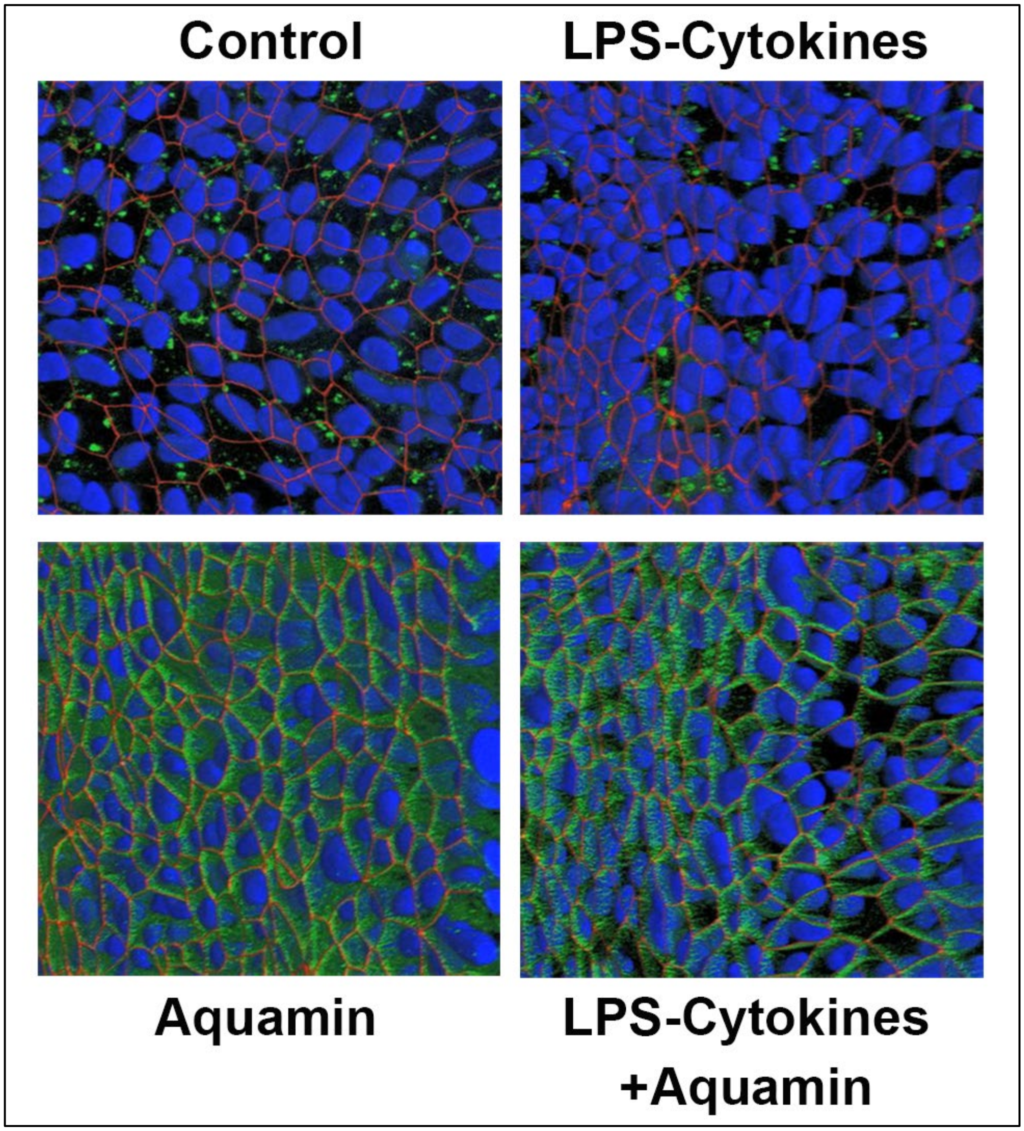

Supplementary Figure 5. Desmoglein-2-stained confocal fluorescent microscopic views from 3D renderings with 45-50° angle to focus on cell surface.

Colon organoids were seeded on transwell membranes to obtain a monolayer and incubated under the specified experimental conditions. Occludin (Red) and desmoglein-2 (green) z-stack images are made up of 40-50 planes per image. DAPI (Blue) was used for nuclear staining. These snapshots are taken from the 3D movie created by using ImageJ Fiji. The images are providing a view of the entire z-stack captured from a 45-50° angle to the cell surface in order to provide a 3-dimensional representation. Occludin (red) staining is apical across all sections. Control conditions show punctate DSG2 staining which is mostly cytoplasmic with little cell surface staining. Desmoglein-2 (green) staining starts apically and extends laterally covering the entire length of the cell surface in the presence of Aquamin® alone and in combination with the pro-inflammatory stimulus. In both confocal images with Aquamin®, a yellowish hue is representing a composite of the two proteins (Occludin and Desmoglein-2).
