# Supplementary material for: A multi-mineral intervention to counter pro-inflammatory activity and to improve the barrier in human colon organoids
Source: Front Cell Dev Biol. 2023 Jul 5;11:1132905. doi: 10.3389/fcell.2023.1132905 (PMC10354648; doi:10.3389/fcell.2023.1132905)
Supplement: Supplementary file 1 [file DataSheet1.zip › Supplementary Movie S1.PDF]

frontiers

Supplementary Material

A Multi-Mineral Intervention to Counter Pro-inflammatory Activity and to Improve the Barrier in Human Colon Organoids

James Varani[1], Shannon D McClintock[1], Daniyal M Nadeem[1], Isabelle Harber[1], Dania Zeidan[1], and Muhammad N Aslam[1*]

*** Correspondence:** Muhammad N Aslam; mnaslam@med.umich.edu

Supplementary Movie (Files) 1.

Available at https://figshare.com/s/761b23c1e5095b0a2e82

Supplementary Movie 1. Confocal Z-stacks generated rotating animation movies.

A 3D rotating representation of occludin and desmoglein-2 staining of organoids derived-epithelial monolayer; a treatment response to control medium (A), LPS-cytokines alone (B), Aquamin® (C), and the combination of LPS-cytokine mix with Aquamin® (D). Confocal-generated Z-stacks were rendered as a 3D movie using Fiji (ImageJ1.52n with BioFormats Importer plugin). Movie rendering from confocal-generated Z-stacks highlights the fact that occludin (red) staining is apical across all sections. Control conditions show punctate DSG2 staining, mostly cytoplasmic with some cell surface staining. Desmoglein-2 (green) staining starts apically and extends laterally covering the entire length of the cell surface in the presence of Aquamin® alone and in combination with the pro-inflammatory stimulus.
