# Supplementary material for: A multi-mineral intervention to counter pro-inflammatory activity and to improve the barrier in human colon organoids
Source: Front Cell Dev Biol. 2023 Jul 5;11:1132905. doi: 10.3389/fcell.2023.1132905 (PMC10354648; doi:10.3389/fcell.2023.1132905)
Supplement: Supplementary file 1 [file DataSheet1.zip › Supplementary Table S10.PDF]

Supplement Table 10A. Down-regulated proteins: The effect of Aquamin alone on the proteomic expression

Proteins	Genes	Treatment Groups<br>**Aquamin**	 LPS-Cytokines	 LPS-Cytokines +Aquamin
Fibrinogen beta chain	FGB	**0.43±0.28***	1.12±0.70	0.53±0.27*
Type-1 angiotensin II receptor-associated protein	AGTRAP	**0.46±0.04***	0.69±0.16*	0.72±0.11*
Fibrinogen gamma chain	FGG	**0.46±0.16*#**	0.89±0.20	0.59±0.24*
CCHC-type zinc finger nucleic acid binding protein	CNBP	**0.48±0.15*#**	1.03±0.30	0.69±0.31
SPARC	SPARC	**0.49±0.06*#**	0.87±0.22	0.54±0.13*
Apolipoprotein C-III	APOC3	**0.50±0.10*#**	1.37±0.35	0.84±0.29
Ribonuclease H2 subunit B	RNASEH2B	**0.52±0.06*#**	1.30±0.34	0.98±0.08
Metallothionein-1H	MT1H	**0.52±0.09*#**	1.37±0.15	2.41±2.72
Translation machinery-associated protein 7	TMA7	**0.55±0.28**	0.82±0.27	0.66±0.02*
Non-histone chromosomal protein HMG-17	HMGN2	**0.56±0.33***	0.86±0.26	0.84±0.19

Values represent average abundance ratio from organoids (n=3 subjects) as compared to the control ± standard deviation. Aquamin alone treatment (**bold**): These proteins were down-regulated at 1.8-fold change (<1% FDR) in response to Aquamin. Corresponding average abundance ratios are provided from the other two treatment groups for comparison. *Represents significance as compared to the control and #represents significance as compared to LPS-Cytokines (at p<0.05).

Supplement Table 10B. Pathways associated with down-regulated proteins altered with Aquamin.

Pathways	Entities pValue	Mapped entities
Signaling by BRAF and RAF1 fusions	2.14×10^{-5}	AGTRAP;FGG;FGB
Oncogenic MAPK signaling	4.37×10^{-5}	AGTRAP;FGG;FGB
p130Cas linkage to MAPK signaling for integrins	7.46×10^{-5}	FGG;FGB
GRB2:SOS provides linkage to MAPK signaling for Integrins	7.46×10^{-5}	FGG;FGB
MyD88 deficiency (TLR2/4)	1.19×10^{-4}	FGG;FGB
IRAK4 deficiency (TLR2/4)	1.32×10^{-4}	FGG;FGB
Regulation of TLR by endogenous ligand	1.46×10^{-4}	FGG;FGB
Platelet degranulation	1.52×10^{-4}	FGG;SPARC;FGB
Common Pathway of Fibrin Clot Formation	1.60×10^{-4}	FGG;FGB
Response to elevated platelet cytosolic Ca2+	1.70×10^{-4}	FGG;SPARC;FGB
Integrin signaling	2.58×10^{-4}	FGG;FGB
Diseases of Immune System	3.80×10^{-4}	FGG;FGB
Diseases associated with the TLR signaling cascade	3.80×10^{-4}	FGG;FGB
Signaling by high-kinase activity BRAF mutants	4.49×10^{-4}	FGG;FGB
Formation of Fibrin Clot (Clotting Cascade)	4.99×10^{-4}	FGG;FGB
Platelet Aggregation (Plug Formation)	5.24×10^{-4}	FGG;FGB
MAP2K and MAPK activation	5.50×10^{-4}	FGG;FGB
Signaling by RAF1 mutants	5.77×10^{-4}	FGG;FGB
Paradoxical activation of RAF signaling by kinase inactive BRAF	7.21×10^{-4}	FGG;FGB

Signaling by moderate kinase activity BRAF mutants	7.21×10^{-4}	FGG;FGB
Signaling downstream of RAS mutants	7.21×10^{-4}	FGG;FGB
Signaling by RAS mutants	7.21×10^{-4}	FGG;FGB
Platelet activation, signaling and aggregation	0.001	FGG;SPARC;FGB
Extracellular matrix organization	0.002	FGG;SPARC;FGB
Integrin cell surface interactions	0.002	FGG;FGB
Scavenging by Class H Receptors	0.003	SPARC
MyD88:MAL(TIRAP) cascade initiated on plasma membrane	0.004	FGG;FGB
Toll Like Receptor TLR6:TLR2 Cascade	0.004	FGG;FGB
Toll Like Receptor TLR1:TLR2 Cascade	0.004	FGG;FGB
Toll Like Receptor 2 (TLR2) Cascade	0.004	FGG;FGB
Diseases of signal transduction by growth factor receptors and second messengers	0.006	AGTRAP;FGG;FGB
Toll Like Receptor 4 (TLR4) Cascade	0.007	FGG;FGB
ER-Phagosome pathway	0.008	FGG;FGB
Chylomicron remodeling	0.009	APOC3
Chylomicron assembly	0.009	APOC3
Toll-like Receptor Cascades	0.009	FGG;FGB
HDL remodeling	0.009	APOC3
Metallothioneins bind metals	0.009	MT1H
Antigen processing-Cross presentation	0.010	FGG;FGB
Response to metal ions	0.012	MT1H
Plasma lipoprotein assembly	0.016	APOC3
Hemostasis	0.021	FGG;SPARC;FGB
RAF/MAP kinase cascade	0.025	FGG;FGB
MAPK1/MAPK3 signaling	0.026	FGG;FGB
Nuclear signaling by ERBB4	0.029	SPARC
Plasma lipoprotein remodeling	0.030	APOC3
MAPK family signaling cascades	0.033	FGG;FGB
Retinoid metabolism and transport	0.037	APOC3
Metabolism of fat-soluble vitamins	0.041	APOC3
Signaling by ERBB4	0.052	SPARC

The pathway analysis report was created by employing Reactome pathway database (v82) for species “Homo sapiens.” The listed pathways were curated by submitting down-regulated proteins altered with Aquamin treatment and presented in Supplement Table 10A.
