# Supplementary material for: A multi-mineral intervention to counter pro-inflammatory activity and to improve the barrier in human colon organoids
Source: Front Cell Dev Biol. 2023 Jul 5;11:1132905. doi: 10.3389/fcell.2023.1132905 (PMC10354648; doi:10.3389/fcell.2023.1132905)
Supplement: Supplementary file 1 [file DataSheet1.zip › Supplementary Table S2.PDF]

Supplement Table 2. Demographic characteristics of subjects providing tissue.

Sample ID	Age	Sex	Ethnicity	Biopsy Site
Colon-87*	21	M	White / caucasian	Ascending colon
Colon-81*	49	F	White / caucasian	Ascending colon
Colon-104	58	F	White / caucasian	Sigmoid colon
Colon-105	62	M	White / caucasian	Sigmoid colon
Colon-106	50	M	White / caucasian	Sigmoid colon

* Source: Gift of Life, Michigan
