# Supplementary material for: A multi-mineral intervention to counter pro-inflammatory activity and to improve the barrier in human colon organoids
Source: Front Cell Dev Biol. 2023 Jul 5;11:1132905. doi: 10.3389/fcell.2023.1132905 (PMC10354648; doi:10.3389/fcell.2023.1132905)
Supplement: Supplementary file 1 [file DataSheet1.zip › Supplementary Table S3.PDF]

Supplement Table 3. Up-regulated proteins: The effect of Aquamin alone on the proteomic expression

Proteins	Genes	Treatment Groups		
		**Aquamin**	LPS-Cytokines	LPS-Cytokines +Aquamin
Keratin, type II cytoskeletal 2 epidermal	KRT2	**8.25±8.80**	0.84±0.51	1.83±1.57
Keratin, type I cytoskeletal 10	KRT10	**7.49±8.18**	0.74±0.59	1.59±0.79
Keratin, type II cytoskeletal 1	KRT1	**5.13±4.52**	0.91±0.68	1.91±0.95
D-dopachrome decarboxylase	DDT	**4.83±5.31**	1.28±0.21	1.04±0.10
ATP-dependent RNA helicase DDX3Y	DDX3Y	**4.00±2.80**	1.73±0.60	3.60±2.06
Plexin-D1	PLXND1	**3.72±2.57**	0.93±0.55	1.83±0.98
Filaggrin-2	FLG2	**3.69±1.71*#**	0.91±0.52	1.80±0.95
Trefoil factor 2	TFF2	**3.28±2.76**	0.79±0.30	1.80±2.00
Dermcidin	DCD	**3.27±1.41*#**	1.00±0.54	3.05±3.12
Annexin A10	ANXA10	**3.27±4.11**	1.07±0.07	4.29±5.97
Alpha-globin transcription factor CP2	TFCP2	**3.22±3.44**	1.00±0.14	1.85±1.33
N-acetylated-alpha-linked acidic dipeptidase 2	NAALAD2	**3.21±3.00**	1.40±0.73	1.50±0.64
Proline-rich protein 15-like protein	PRR15L	**3.01±2.73**	1.29±0.22	1.80±0.60
Kallikrein-7	KLK7	**2.56±1.26***	1.17±0.23	4.70±5.22
Ankyrin repeat and SAM domain-containing protein 1A	ANKS1A	**2.46±2.77**	1.50±0.77	1.46±0.73
Glutathione S-transferase A1	GSTA1	**2.45±1.87**	0.56±0.24*	2.42±3.12
Phosphofurin acidic cluster sorting protein 1	PACS1	**2.44±2.03**	1.24±0.48	1.24±0.27
Cadherin-17	CDH17	**2.44±0.32*#**	0.71±0.06*	2.07±0.12*#
Melanotransferrin	MELTF	**2.43±1.42**	1.44±0.33*	3.91±3.53
Leucine zipper putative tumor suppressor 1	LZTS1	**2.42±2.11**	1.13±0.38	1.49±0.67
Keratin, type I cytoskeletal 9	KRT9	**2.42±1.05***	1.19±0.78	2.20±1.02
Aldo-keto reductase family 1 member C2	AKR1C2	**2.41±1.33**	0.78±0.05*	1.11±0.53
Keratin, type II cytoskeletal 5	KRT5	**2.36±1.58**	0.89±0.51	1.37±0.61
HLA class I histocompatibility antigen, alpha chain G	HLA-G	**2.32±2.29**	2.03±0.35*	2.30±0.08*
Carbonic anhydrase 1	CA1	**2.29±2.30**	0.97±0.10	1.02±0.32
Clarin-3	CLRN3	**2.28±1.08**	1.11±0.25	1.26±0.25
Ly6/PLAUR domain-containing protein 8	LYPD8	**2.24±1.08#**	0.68±0.31	0.84±0.54
Tumor necrosis factor alpha-induced protein 2	TNFAIP2	**2.24±2.10**	1.90±1.61	2.42±1.88
Keratin, type II cytoskeletal 80	KRT80	**2.24±1.49**	1.31±0.99	1.10±0.39
Keratin, type II cytoskeletal 1b	KRT77	**2.23±1.25**	0.61±0.30	1.10±0.38
Golgi to ER traffic protein 4 homolog	GET4	**2.22±2.11**	1.30±0.22*	1.17±0.31
HLA class II histocompatibility antigen, DR beta 5 chain	HLA-DRB5	**2.17±0.00**	18.83±15.19	7.49±7.14
Dual oxidase 2	DUOX2	**2.16±0.63***	1.89±1.35	2.47±1.06*
Olfactomedin-4	OLFM4	**2.15±1.67**	0.76±0.34	2.16±2.10
Calcium-activated chloride channel regulator 4	CLCA4	**2.14±1.26**	1.42±0.72	1.40±1.02
Serine/threonine-protein kinase 25	STK25	**2.12±1.26**	1.55±0.48	1.81±0.66
ETS homologous factor	EHF	**2.12±2.18**	1.17±0.28	1.44±0.66
Protein adenylyltransferase SelO, mitochondrial	SELENOO	**2.11±2.29**	1.18±0.44	1.48±1.16
Eukaryotic translation initiation factor 2D	EIF2D	**2.10±1.26**	1.14±0.10	1.45±0.54

Pantetheinase	VNN1	**2.08±1.83**	1.44±0.90	1.68±0.94
Sulfotransferase 1A4	SULT1A4	**2.06±1.79**	0.97±0.13	1.32±0.69
Sulfotransferase 1B1	SULT1B1	**2.06±1.71**	0.83±0.29	1.35±0.98
Xaa-Pro dipeptidase	PEPD	**2.05±1.49**	1.07±0.25	1.49±0.90
15-hydroxyprostaglandin dehydrogenase [NAD(+)]	HPGD	**2.05±0.69*#**	1.00±0.16	1.50±0.61
AT-rich interactive domain-containing protein 1B	ARID1B	**2.04±1.39**	1.07±0.21	1.32±0.46
Protein lifeguard 3	TMBIM1	**2.03±1.71**	1.32±0.27	1.97±1.82
Indian hedgehog protein	IHH	**2.02±1.05**	0.96±0.18	0.93±0.09
Gasdermin-B	GSDMB	**2.01±1.25**	2.34±0.23*	2.02±0.32*
Medium-chain acyl-CoA ligase ACSF2, mitochondrial	ACSF2	**2.01±0.72*#**	0.98±0.12	1.38±0.36
Protocadherin-1	PCDH1	**1.98±0.41*#**	0.88±0.10*	1.80±0.14*#
Phenazine biosynthesis-like domain-containing protein	PBLD	**1.95±0.62*#**	0.87±0.08*	1.63±0.29*#
Keratin, type I cytoskeletal 16	KRT16	**1.95±1.12**	1.07±1.07	2.00±1.53
Iron-sulfur protein NUBPL	NUBPL	**1.94±0.99**	1.10±0.05*	1.47±0.27*
Keratin, type I cytoskeletal 14	KRT14	**1.93±1.03**	0.70±0.57	0.97±0.18
UDP-N-acetylglucosamine--peptide N-acetylglucosaminyltransferase 110 kDa subunit	OGT	**1.92±1.71**	1.26±0.20*	1.40±0.40
Receptor-type tyrosine-protein phosphatase eta	PTPRJ	**1.92±0.46***	1.26±0.10*	1.23±0.44
Very long-chain acyl-CoA synthetase	SLC27A2	**1.91±1.16**	0.93±0.10	1.29±0.30
Interferon-induced, double-stranded RNA-activated protein kinase	EIF2AK2	**1.90±1.90**	1.51±0.02*	1.87±0.92
Kallikrein-6	KLK6	**1.89±0.54*#**	0.88±0.08*	2.43±1.86
Prostate stem cell antigen	PSCA	**1.88±0.88#**	0.62±0.33	1.06±0.53
Transmembrane 4 L6 family member 1	TM4SF1	**1.88±1.42**	1.27±0.41	1.10±0.12
Thymidine kinase 2, mitochondrial	TK2	**1.88±1.15**	1.01±0.40	1.13±0.37
Legumain	LGMN	**1.87±0.40***	1.58±0.10*	2.28±0.47*#
Cytochrome c oxidase subunit 1	COX1	**1.87±1.64**	1.23±0.24	1.25±0.41
Desmoglein-2	DSG2	**1.85±0.43*#**	0.97±0.09	1.80±0.08*#
Rhomboid-related protein 2	RHBDL2	**1.85±0.02***	1.18±0.58	1.07±0.52
Chloride anion exchanger	SLC26A3	**1.85±0.19*#**	1.05±0.34	0.97±0.68
Phosphoenolpyruvate carboxykinase, cytosolic [GTP]	PCK1	**1.84±1.02#**	0.55±0.24*	1.10±0.42
Carcinoembryonic antigen-related cell adhesion molecule 5	CEACAM5	**1.84±1.38**	1.29±0.29	1.24±0.26
FERM, ARHGEF and pleckstrin domain-containing protein 2	FARP2	**1.84±1.21**	1.01±0.16	1.37±0.61
Zinc finger protein 638	ZNF638	**1.83±2.05**	1.17±0.24	1.09±0.43
Podocalyxin	PODXL	**1.83±0.69**	1.22±0.18	1.38±0.40
Gasdermin-D	GSDMD	**1.82±0.94**	2.15±0.37*	2.98±1.05*
Microtubule-associated protein RP/EB family member 2	MAPRE2	**1.80±0.91**	0.95±0.13	1.21±0.35
UBX domain-containing protein 6	UBXN6	**1.80±1.43**	1.04±0.28	1.27±0.43
Forkhead box protein K1	FOXK1	**1.80±1.93**	1.21±0.43	1.37±0.95
STE20/SPS1-related proline-alanine-rich protein kinase	STK39	**1.80±1.45**	1.03±0.11	1.40±0.46
Atypical kinase COQ8A, mitochondrial	COQ8A	**1.80±0.39*#**	0.82±0.18	1.07±0.24
Cytochrome c	CYCS	**1.80±1.26**	1.10±0.10	1.07±0.26

Indoleamine 2,3-dioxygenase 1	IDO1	**1.80±0.81**	9.60±4.81*	5.59±5.08
Pyridoxal-dependent decarboxylase domain-containing protein 1	PDXDC1	**1.80±1.22**	1.04±0.11	1.33±0.46

Values represent average abundance ratio from organoids (n=3 subjects) as compared to the control ± standard deviation. Aquamin alone treatment (**bold**): These proteins were up-regulated at 1.8-fold change (<1% FDR except NAALAD2 and TK2 which are <2%FDR) in response to Aquamin. Corresponding average abundance ratios are provided from the other two treatment groups for comparison. *Represents significance as compared to the control and #represents significance as compared to LPS-Cytokines (at $p<0.05$).
