# Supplementary material for: A multi-mineral intervention to counter pro-inflammatory activity and to improve the barrier in human colon organoids
Source: Front Cell Dev Biol. 2023 Jul 5;11:1132905. doi: 10.3389/fcell.2023.1132905 (PMC10354648; doi:10.3389/fcell.2023.1132905)
Supplement: Supplementary file 1 [file DataSheet1.zip › Supplementary Table S6.PDF]

Supplement Table 6. Up-regulated proteins: The effect of LPS-Cytokines on the proteomic expression

		Treatment Groups		
Proteins	Genes	**LPS-Cytokines**	LPS-Cytokines +Aquamin	Aquamin
HLA class II histocompatibility antigen, DP alpha 1 chain	HLA-DPA1	**45.09±38.24**	42.83±35.69	1.48±0.71
HLA class II histocompatibility antigen, DR beta 5 chain	HLA-DRB5	**18.83±15.19**	7.49±7.14	2.17±0.00
HLA class II histocompatibility antigen, DR alpha chain	HLA-DRA	**16.35±3.16***	13.85±3.85*	1.15±0.14#
HLA class II histocompatibility antigen, DR beta 3 chain	HLA-DRB3	**13.51±4.30***	9.82±5.95	1.05±0.07#
HLA class II histocompatibility antigen, DRB1 beta chain	HLA-DRB1	**11.58±2.89***	9.65±1.21*	1.03±0.05#
Protein-glutamine gamma-glutamyltransferase 2	TGM2	**10.26±8.41**	8.37±0.51*	1.39±0.31*
Tryptophan--tRNA ligase, cytoplasmic	WARS	**9.88±1.23***	7.11±1.36*	1.02±0.10#
Indoleamine 2,3-dioxygenase 1	IDO1	**9.60±4.81***	5.59±5.08	1.77±0.81
HLA class II histocompatibility antigen gamma chain	CD74	**7.90±1.19***	7.73±0.87*	1.19±0.11*#
HLA class II histocompatibility antigen, DP beta 1 chain	HLA-DPB1	**6.65±5.71**	3.90±0.38*	0.90±0.21
HLA class I histocompatibility antigen, B alpha chain	HLA-B	**6.56±1.44***	4.41±3.11	1.07±0.52#
Caspase-1	CASP1	**5.90±3.07**	3.24±0.79*	1.45±0.54
Phospholipase A2, membrane associated	PLA2G2A	**5.60±1.11***	1.74±1.81#	0.72±0.25#
Signal transducer and activator of transcription 1-alpha/beta	STAT1	**5.36±0.67***	4.96±1.52*	1.20±0.48#
Interferon-induced GTP-binding protein Mx1	MX1	**5.30±0.97***	5.26±1.16*	1.25±0.33#
HLA class I histocompatibility antigen, alpha chain F	HLA-F	**4.95±1.39***	4.25±0.32*	1.22±0.26#
Guanylate-binding protein 1	GBP1	**4.77±0.90***	4.21±1.14*	1.02±0.05#
Ubiquitin/ISG15-conjugating enzyme E2 L6	UBE2L6	**4.74±4.03**	2.34±0.48*	1.14±0.05*
Zinc finger MYM-type protein 2	ZMYM2	**4.73±3.39**	9.37±2.88*	1.04±0.29
HLA class II histocompatibility antigen, DM beta chain	HLA-DMB	**4.34±0.19***	3.41±0.97*	0.99±0.17#
Tumor necrosis factor receptor superfamily member 5	CD40	**4.26±1.91***	4.11±2.75	0.95±0.23#
Proteasome subunit beta type-9	PSMB9	**4.26±1.28***	2.97±1.12*	1.12±0.42#
Thymidine phosphorylase	TYMP	**4.10±1.44***	4.07±1.30*	1.15±0.17#
Antigen peptide transporter 1	TAP1	**4.09±0.95***	3.76±0.76*	1.05±0.18#
Interferon-induced transmembrane protein 2	IFITM2	**4.06±1.83***	5.34±4.72	0.82±0.39#
HLA class I histocompatibility antigen, A alpha chain	HLA-A	**4.05±0.65***	3.98±0.58*	1.07±0.13#
Cytosol aminopeptidase	LAP3	**3.89±1.18***	2.82±0.27*	1.07±0.12#
HLA class II histocompatibility antigen, DR beta 4 chain	HLA-DRB4	**3.81±2.35**	3.20±1.37	1.34±0.03*
Apolipoprotein L2	APOL2	**3.78±0.83***	3.53±0.34*	1.46±0.52#
Beta-2-microglobulin	B2M	**3.68±0.43***	3.74±1.65*	1.04±0.13#
HLA class I histocompatibility antigen, C alpha chain	HLA-C	**3.68±0.47***	3.90±0.80*	1.04±0.19#

Antigen peptide transporter 2	TAP2	**3.61±0.34***	3.63±0.38*	1.06±0.14#
Interleukin-32	IL32	**3.61±2.18**	3.48±1.79*	1.17±0.13*
Proteasome subunit beta type-10	PSMB10	**3.56±0.62***	2.44±0.06*#	1.15±0.19#
Cathepsin L2	CTSV	**3.40±1.14***	3.32±1.62	1.44±0.22*#
Intercellular adhesion molecule 1	ICAM1	**3.13±1.34**	2.38±1.70	0.79±0.07#
Complement factor B	CFB	**3.13±0.47***	3.49±0.44*	1.42±0.30*#
Nitric oxide synthase, inducible	NOS2	**3.07±1.10***	3.79±0.17*	1.10±0.15#
Tapasin	TAPBP	**3.07±0.54***	3.25±0.88*	0.92±0.08#
Retinoic acid receptor responder protein 1	RARRES1	**3.03±0.77***	3.15±0.27*	1.14±0.08*#
Butyrophilin subfamily 3 member A3	BTN3A3	**2.97±1.44***	3.05±2.07	1.11±0.20#
Gamma-interferon-inducible lysosomal thiol reductase	IFI30	**2.90±0.63***	3.22±0.37*	1.05±0.22#
Sterile alpha motif domain-containing protein 9-like	SAMD9L	**2.86±0.79***	2.35±0.49*	1.39±0.31*#
2'-5'-oligoadenylate synthase 3	OAS3	**2.85±0.49***	2.63±1.07*	1.08±0.46#
HLA class I histocompatibility antigen, alpha chain E	HLA-E	**2.82±0.65***	2.29±0.40*	1.46±0.41#
Midkine	MDK	**2.67±0.77***	1.88±0.19*	1.31±0.80
Guanylate-binding protein 2	GBP2	**2.65±0.20***	2.38±0.32*	1.10±0.17#
Ubiquitin-like protein ISG15	ISG15	**2.58±0.57***	2.19±0.39*	1.25±0.18*#
Proteasome activator complex subunit 2	PSME2	**2.58±0.37***	2.42±0.36*	1.09±0.12#
Ceruloplasmin	CP	**2.45±2.05**	3.44±2.03	1.41±0.77
Alpha-1-antitrypsin	SERPINA1	**2.44±0.72***	2.76±1.35*	1.40±0.29*#
Antiviral innate immune response receptor RIG-I	DDX58	**2.35±0.84***	2.12±0.69*	0.93±0.27#
Plasminogen activator inhibitor 2	SERPINB2	**2.34±1.02**	2.73±1.25	1.24±0.43
Gasdermin-B	GSDMB	**2.34±0.23***	2.02±0.32*	2.01±1.25
Visinin-like protein 1	VSNL1	**2.28±0.57***	2.53±1.15*	1.22±0.32#
UMP-CMP kinase 2, mitochondrial	CMPK2	**2.27±0.99**	2.20±1.15	0.99±0.33
Protein mono-ADP-ribosyltransferase PARP9	PARP9	**2.24±0.37***	2.36±0.38*	1.17±0.50#
Proteasome activator complex subunit 1	PSME1	**2.24±0.45***	2.09±0.43*	1.19±0.17#
Mucin-1	MUC1	**2.23±0.79***	2.26±1.07	1.42±0.55
Probable ATP-dependent RNA helicase DDX60	DDX60	**2.22±0.28***	2.66±0.87*	1.44±0.29*#
Gasdermin-D	GSDMD	**2.15±0.37***	2.98±1.05*	1.82±0.94
F-box only protein 6	FBXO6	**2.15±0.52***	2.15±0.74*	1.01±0.14#
Tapasin-related protein	TAPBPL	**2.14±0.50***	2.28±0.77*	1.53±0.60
E3 ubiquitin-protein ligase RNF213	RNF213	**2.12±0.09***	1.87±0.61*	1.14±0.54#
Interferon-induced protein with tetratricopeptide repeats 5	IFIT5	**2.10±1.06**	1.67±1.01	1.09±0.24
Integral membrane protein 2B	ITM2B	**2.09±0.73***	1.60±0.22*	1.17±0.08*#
Protein mono-ADP-ribosyltransferase PARP14	PARP14	**2.04±0.39***	1.75±0.52	1.12±0.33#
HLA class I histocompatibility antigen, alpha chain G	HLA-G	**2.03±0.35***	2.30±0.08*	2.32±2.29
Argininosuccinate synthase	ASS1	**1.99±0.15***	1.64±0.65	0.99±0.22#
Ubiquitin-like modifier-activating enzyme 7	UBA7	**1.92±0.35***	2.17±0.33*	1.29±0.39
E3 ubiquitin-protein ligase TRIM21	TRIM21	**1.91±0.45***	1.86±0.55*	0.91±0.21#
CWF19-like protein 1	CWF19L1	**1.91±1.44**	1.11±0.01*#	1.22±0.04*
Cathepsin S	CTSS	**1.91±0.20***	1.62±0.69	1.22±0.23#

Tumor necrosis factor alpha-induced protein 2	TNFAIP2	**1.90±1.61**	2.42±1.88	2.24±2.10
Dual oxidase 2	DUOX2	**1.89±1.35**	2.47±1.06*	2.16±0.63*
Caspase-7	CASP7	**1.88±0.27***	1.93±0.15*	1.22±0.26#
Leukocyte surface antigen CD47	CD47	**1.87±0.08***	1.62±0.20*	0.93±0.30#
Chloride intracellular channel protein 5	CLIC5	**1.86±0.35***	2.16±0.23*	1.33±0.31
NEDD8 ultimate buster 1	NUB1	**1.86±0.81**	1.46±0.06*	1.03±0.12
CD82 antigen	CD82	**1.85±0.41***	2.20±1.24	1.28±0.88
Caspase-10	CASP10	**1.84±0.51***	1.50±0.14*	1.28±0.40
Ferritin heavy chain	FTH1	**1.83±0.21***	1.24±0.21#	0.83±0.12*#
Mitochondrial glutamate carrier 1	SLC25A22	**1.81±0.09***	1.79±0.30*	1.12±0.30#
Shiftless antiviral inhibitor of ribosomal frameshifting protein	SHFL	**1.80±0.29***	1.82±0.47*	1.22±0.44
Gamma-interferon-inducible protein 16	IFI16	**1.80±0.74**	1.95±0.80	1.30±0.27
2'-5'-oligoadenylate synthase 1	OAS1	**1.80±0.20***	3.01±1.52	1.37±0.38

Values represent average abundance ratio from organoids (n=3 subjects) as compared to the control ± standard deviation. LPS-cytokine treatment (**bold**): These proteins were up-regulated at 1.8-fold change (<1% FDR) in response to LPS-Cytokine mix. Corresponding average abundance ratios are provided from the other two treatment groups for comparison. *Represents significance as compared to the control and #represents significance as compared to LPS-Cytokines (at $p<0.05$).
