# Supplementary material for: A multi-mineral intervention to counter pro-inflammatory activity and to improve the barrier in human colon organoids
Source: Front Cell Dev Biol. 2023 Jul 5;11:1132905. doi: 10.3389/fcell.2023.1132905 (PMC10354648; doi:10.3389/fcell.2023.1132905)
Supplement: Supplementary file 1 [file DataSheet1.zip › Supplementary Table S8.PDF]

Supplement Table 8. Pathways associated with down-regulated proteins altered with LPS-Cytokines.

Pathways	Entities pValue	Mapped entities
Nuclear events mediated by NFE2L2	1.47×10^{-7}	PSMB7;GSTA1;PSMB5;PSMB6
KEAP1-NFE2L2 pathway	4.21×10^{-7}	PSMB7;GSTA1;PSMB5;PSMB6
Regulation of activated PAK-2p34 by proteasome mediated degradation	4.40×10^{-6}	PSMB7;PSMB5;PSMB6
Cross-presentation of soluble exogenous antigens (endosomes)	4.40×10^{-6}	PSMB7;PSMB5;PSMB6
Regulation of ornithine decarboxylase (ODC)	4.67×10^{-6}	PSMB7;PSMB5;PSMB6
p53-Independent G1/S DNA damage checkpoint	4.94×10^{-6}	PSMB7;PSMB5;PSMB6
GSK3B and BTRC:CUL1-mediated-degradation of NFE2L2	4.94×10^{-6}	PSMB7;PSMB5;PSMB6
Ubiquitin Mediated Degradation of Phosphorylated Cdc25A	4.94×10^{-6}	PSMB7;PSMB5;PSMB6
p53-Independent DNA Damage Response	4.94×10^{-6}	PSMB7;PSMB5;PSMB6
Autodegradation of the E3 ubiquitin ligase COP1	4.94×10^{-6}	PSMB7;PSMB5;PSMB6
Ubiquitin-dependent degradation of Cyclin D	4.94×10^{-6}	PSMB7;PSMB5;PSMB6
Vpu mediated degradation of CD4	5.23×10^{-6}	PSMB7;PSMB5;PSMB6
Regulation of Apoptosis	5.23×10^{-6}	PSMB7;PSMB5;PSMB6
Cellular response to chemical stress	5.38×10^{-6}	PSMB7;GSTA1;PSMB5;PSMB6
FBXL7 down-regulates AURKA during mitotic entry and in early mitosis	5.84×10^{-6}	PSMB7;PSMB5;PSMB6
SCF-beta-TrCP mediated degradation of Emi1	5.84×10^{-6}	PSMB7;PSMB5;PSMB6
Degradation of AXIN	5.84×10^{-6}	PSMB7;PSMB5;PSMB6
Negative regulation of NOTCH4 signaling	5.84×10^{-6}	PSMB7;PSMB5;PSMB6
Regulation of RUNX3 expression and activity	5.84×10^{-6}	PSMB7;PSMB5;PSMB6
AUF1 (hnRNP D0) binds and destabilizes mRNA	6.17×10^{-6}	PSMB7;PSMB5;PSMB6
Vif-mediated degradation of APOBEC3G	6.17×10^{-6}	PSMB7;PSMB5;PSMB6
Hh mutants are degraded by ERAD	6.17×10^{-6}	PSMB7;PSMB5;PSMB6
Degradation of DVL	6.50×10^{-6}	PSMB7;PSMB5;PSMB6
Stabilization of p53	6.50×10^{-6}	PSMB7;PSMB5;PSMB6
Hh mutants abrogate ligand secretion	7.20×10^{-6}	PSMB7;PSMB5;PSMB6
NIK-->noncanonical NF-kB signaling	7.20×10^{-6}	PSMB7;PSMB5;PSMB6
Metabolism of polyamines	7.20×10^{-6}	PSMB7;PSMB5;PSMB6
Degradation of GLI1 by the proteasome	7.57×10^{-6}	PSMB7;PSMB5;PSMB6
GLI3 is processed to GLI3R by the proteasome	7.57×10^{-6}	PSMB7;PSMB5;PSMB6
Degradation of GLI2 by the proteasome	7.57×10^{-6}	PSMB7;PSMB5;PSMB6
SCF(Skp2)-mediated degradation of p27/p21	7.57×10^{-6}	PSMB7;PSMB5;PSMB6
Dectin-1 mediated noncanonical NF-kB signaling	7.57×10^{-6}	PSMB7;PSMB5;PSMB6
Defective CFTR causes cystic fibrosis	7.96×10^{-6}	PSMB7;PSMB5;PSMB6
Autodegradation of Cdh1 by Cdh1:APC/C	9.18×10^{-6}	PSMB7;PSMB5;PSMB6
Asymmetric localization of PCP proteins	9.18×10^{-6}	PSMB7;PSMB5;PSMB6
Hedgehog ligand biogenesis	9.62×10^{-6}	PSMB7;PSMB5;PSMB6
Oxygen-dependent proline hydroxylation of Hypoxia-inducible Factor Alpha	1.01×10^{-5}	PSMB7;PSMB5;PSMB6
p53-Dependent G1 DNA Damage Response	1.01×10^{-5}	PSMB7;PSMB5;PSMB6
p53-Dependent G1/S DNA damage checkpoint	1.01×10^{-5}	PSMB7;PSMB5;PSMB6
Activation of NF-kappaB in B cells	1.05×10^{-5}	PSMB7;PSMB5;PSMB6
APC/C:Cdc20 mediated degradation of Securin	1.10×10^{-5}	PSMB7;PSMB5;PSMB6
G1/S DNA Damage Checkpoints	1.10×10^{-5}	PSMB7;PSMB5;PSMB6
Regulation of RAS by GAPs	1.15×10^{-5}	PSMB7;PSMB5;PSMB6

Regulation of PTEN stability and activity	1.15×10^{-5}	PSMB7;PSMB5;PSMB6
Orc1 removal from chromatin	1.25×10^{-5}	PSMB7;PSMB5;PSMB6
Cdc20:Phospho-APC/C mediated degradation of Cyclin A	1.36×10^{-5}	PSMB7;PSMB5;PSMB6
CDK-mediated phosphorylation and removal of Cdc6	1.36×10^{-5}	PSMB7;PSMB5;PSMB6
APC/C:Cdh1 mediated degradation of Cdc20 and other APC/C:Cdh1 targeted proteins in late mitosis/early G1	1.41×10^{-5}	PSMB7;PSMB5;PSMB6
APC:Cdc20 mediated degradation of cell cycle proteins prior to satisfation of the cell cycle checkpoint	1.41×10^{-5}	PSMB7;PSMB5;PSMB6
Regulation of RUNX2 expression and activity	1.41×10^{-5}	PSMB7;PSMB5;PSMB6
APC/C:Cdc20 mediated degradation of mitotic proteins	1.53×10^{-5}	PSMB7;PSMB5;PSMB6
Cellular response to hypoxia	1.53×10^{-5}	PSMB7;PSMB5;PSMB6
Activation of APC/C and APC/C:Cdc20 mediated degradation of mitotic proteins	1.59×10^{-5}	PSMB7;PSMB5;PSMB6
ABC transporter disorders	1.65×10^{-5}	PSMB7;PSMB5;PSMB6
The role of GTSE1 in G2/M progression after G2 checkpoint	1.72×10^{-5}	PSMB7;PSMB5;PSMB6
Regulation of APC/C activators between G1/S and early anaphase	1.85×10^{-5}	PSMB7;PSMB5;PSMB6
Downstream signaling events of B Cell Receptor (BCR)	1.92×10^{-5}	PSMB7;PSMB5;PSMB6
Cyclin E associated events during G1/S transition	1.99×10^{-5}	PSMB7;PSMB5;PSMB6
Signaling by NOTCH4	1.99×10^{-5}	PSMB7;PSMB5;PSMB6
Degradation of beta-catenin by the destruction complex	1.99×10^{-5}	PSMB7;PSMB5;PSMB6
Hedgehog 'on' state	2.14×10^{-5}	PSMB7;PSMB5;PSMB6
Cyclin A:Cdk2-associated events at S phase entry	2.14×10^{-5}	PSMB7;PSMB5;PSMB6
Regulation of mitotic cell cycle	2.37×10^{-5}	PSMB7;PSMB5;PSMB6
APC/C-mediated degradation of cell cycle proteins	2.37×10^{-5}	PSMB7;PSMB5;PSMB6
Regulation of mRNA stability by proteins that bind AU-rich elements	2.37×10^{-5}	PSMB7;PSMB5;PSMB6
MAPK6/MAPK4 signaling	2.45×10^{-5}	PSMB7;PSMB5;PSMB6
Switching of origins to a post-replicative state	2.70×10^{-5}	PSMB7;PSMB5;PSMB6
PCP/CE pathway	2.70×10^{-5}	PSMB7;PSMB5;PSMB6
UCH proteinases	3.07×10^{-5}	PSMB7;PSMB5;PSMB6
Transcriptional regulation by RUNX3	3.07×10^{-5}	PSMB7;PSMB5;PSMB6
CLEC7A (Dectin-1) signaling	3.26×10^{-5}	PSMB7;PSMB5;PSMB6
RUNX1 regulates transcription of genes involved in differentiation of HSCs	3.36×10^{-5}	PSMB7;PSMB5;PSMB6
TNFR2 non-canonical NF-kB pathway	3.67×10^{-5}	PSMB7;PSMB5;PSMB6
ABC-family proteins mediated transport	3.78×10^{-5}	PSMB7;PSMB5;PSMB6
Assembly of the pre-replicative complex	4.84×10^{-5}	PSMB7;PSMB5;PSMB6
Hedgehog 'off' state	5.11×10^{-5}	PSMB7;PSMB5;PSMB6
Interleukin-1 signaling	5.24×10^{-5}	PSMB7;PSMB5;PSMB6
Downstream TCR signaling	5.38×10^{-5}	PSMB7;PSMB5;PSMB6
Synthesis of DNA	6.09×10^{-5}	PSMB7;PSMB5;PSMB6
Transcriptional regulation by RUNX2	6.09×10^{-5}	PSMB7;PSMB5;PSMB6
DNA Replication Pre-Initiation	7.36×10^{-5}	PSMB7;PSMB5;PSMB6
G1/S Transition	7.70×10^{-5}	PSMB7;PSMB5;PSMB6
TCR signaling	8.99×10^{-5}	PSMB7;PSMB5;PSMB6
PTEN Regulation	9.18×10^{-5}	PSMB7;PSMB5;PSMB6
Host Interactions of HIV factors	1.02×10^{-4}	PSMB7;PSMB5;PSMB6
Beta-catenin independent WNT signaling	1.02×10^{-4}	PSMB7;PSMB5;PSMB6
Mitotic G1 phase and G1/S transition	1.13×10^{-4}	PSMB7;PSMB5;PSMB6
Signaling by Hedgehog	1.15×10^{-4}	PSMB7;PSMB5;PSMB6
G2/M Checkpoints	1.15×10^{-4}	PSMB7;PSMB5;PSMB6

Interleukin-1 family signaling	1.27×10^{-4}	PSMB7;PSMB5;PSMB6
DNA Replication	1.32×10^{-4}	PSMB7;PSMB5;PSMB6
ER-Phagosome pathway	1.39×10^{-4}	PSMB7;PSMB5;PSMB6
S Phase	1.47×10^{-4}	PSMB7;PSMB5;PSMB6
FCERI mediated NF-kB activation	1.58×10^{-4}	PSMB7;PSMB5;PSMB6
Regulation of expression of SLITs and ROBOs	1.72×10^{-4}	PSMB7;PSMB5;PSMB6
C-type lectin receptors (CLRs)	1.78×10^{-4}	PSMB7;PSMB5;PSMB6
Antigen processing-Cross presentation	1.84×10^{-4}	PSMB7;PSMB5;PSMB6
Signaling by the B Cell Receptor (BCR)	1.84×10^{-4}	PSMB7;PSMB5;PSMB6
Disorders of transmembrane transporters	2.00×10^{-4}	PSMB7;PSMB5;PSMB6
Apoptosis	2.03×10^{-4}	PSMB7;PSMB5;PSMB6
Separation of Sister Chromatids	2.34×10^{-4}	PSMB7;PSMB5;PSMB6
G2/M Transition	2.60×10^{-4}	PSMB7;PSMB5;PSMB6
Mitotic G2-G2/M phases	2.68×10^{-4}	PSMB7;PSMB5;PSMB6
TCF dependent signaling in response to WNT	2.76×10^{-4}	PSMB7;PSMB5;PSMB6
Ub-specific processing proteases	2.88×10^{-4}	PSMB7;PSMB5;PSMB6
Signaling by NOTCH	2.92×10^{-4}	PSMB7;PSMB5;PSMB6
Transcriptional regulation by RUNX1	3.01×10^{-4}	PSMB7;PSMB5;PSMB6
Programmed Cell Death	3.41×10^{-4}	PSMB7;PSMB5;PSMB6
Signaling by ROBO receptors	3.45×10^{-4}	PSMB7;PSMB5;PSMB6
Fc epsilon receptor (FCERI) signaling	3.45×10^{-4}	PSMB7;PSMB5;PSMB6
Developmental Biology	3.62×10^{-4}	PSMB7;PI3;PSMB5;PSMB6;PCK1
Mitotic Anaphase	4.41×10^{-4}	PSMB7;PSMB5;PSMB6
Mitotic Metaphase and Anaphase	4.46×10^{-4}	PSMB7;PSMB5;PSMB6
Neddylation	4.74×10^{-4}	PSMB7;PSMB5;PSMB6
HIV Infection	4.86×10^{-4}	PSMB7;PSMB5;PSMB6
Innate Immune System	5.06×10^{-4}	PSMB7;SLPI;PI3;PSMB5;PSMB6
Cell Cycle Checkpoints	6.73×10^{-4}	PSMB7;PSMB5;PSMB6
PIP3 activates AKT signaling	7.24×10^{-4}	PSMB7;PSMB5;PSMB6
Deubiquitination	7.39×10^{-4}	PSMB7;PSMB5;PSMB6
RAF/MAP kinase cascade	8.10×10^{-4}	PSMB7;PSMB5;PSMB6
Signaling by WNT	8.67×10^{-4}	PSMB7;PSMB5;PSMB6
MAPK1/MAPK3 signaling	8.67×10^{-4}	PSMB7;PSMB5;PSMB6
Antigen processing: Ubiquitination & Proteasome degradation	9.54×10^{-4}	PSMB7;PSMB5;PSMB6
Cellular responses to stress	0.001	PSMB7;GSTA1;PSMB5;PSMB6
Intracellular signaling by second messengers	0.001	PSMB7;PSMB5;PSMB6
Cellular responses to stimuli	0.001	PSMB7;GSTA1;PSMB5;PSMB6
MAPK family signaling cascades	0.001	PSMB7;PSMB5;PSMB6
Metabolism of amino acids and derivatives	0.002	PSMB7;PSMB5;PSMB6
M Phase	0.002	PSMB7;PSMB5;PSMB6
Drug ADME	0.002	GSTA1;PCK1
Class I MHC mediated antigen processing & presentation	0.003	PSMB7;PSMB5;PSMB6
Diseases of signal transduction by growth factor receptors and second messengers	0.003	PSMB7;PSMB5;PSMB6
Signaling by Interleukins	0.003	PSMB7;PSMB5;PSMB6
NR1H2 & NR1H3 regulate gene expression linked to gluconeogenesis	0.003	PCK1
Abacavir metabolism	0.003	PCK1
Cell Cycle, Mitotic	0.005	PSMB7;PSMB5;PSMB6
Axon guidance	0.005	PSMB7;PSMB5;PSMB6
Nervous system development	0.006	PSMB7;PSMB5;PSMB6

Generic Transcription Pathway	0.007	PSMB7;PSMB5;PSMB6;PCK1
Abacavir ADME	0.007	PCK1
Metabolism	0.007	PSMB7;GSTA1;PSMB5;PSMB6;PCK1
Cell Cycle	0.009	PSMB7;PSMB5;PSMB6
Metabolism of RNA	0.009	PSMB7;PSMB5;PSMB6
Immune System	0.009	PSMB7;SLPI;PI3;PSMB5;PSMB6
RNA Polymerase II Transcription	0.009	PSMB7;PSMB5;PSMB6;PCK1
Heme degradation	0.011	GSTA1
Transport of small molecules	0.011	PSMB7;PSMB5;PSMB6
Gene expression (Transcription)	0.013	PSMB7;PSMB5;PSMB6;PCK1
Cytokine Signaling in Immune system	0.015	PSMB7;PSMB5;PSMB6
Azathioprine ADME	0.016	GSTA1
Signal Transduction	0.017	RGS16;PSMB7;PSMB5;PSMB6;PCK1
Metabolism of porphyrins	0.020	GSTA1
FOXO-mediated transcription of oxidative stress, metabolic and neuronal genes	0.020	PCK1
Adaptive Immune System	0.023	PSMB7;PSMB5;PSMB6
Gluconeogenesis	0.023	PCK1
Glutathione conjugation	0.025	GSTA1
NR1H2 and NR1H3-mediated signaling	0.033	PCK1
G alpha (z) signalling events	0.033	RGS16
Neutrophil degranulation	0.041	PSMB7;SLPI
FOXO-mediated transcription	0.045	PCK1

The pathway analysis report was created by employing Reactome pathway database (v82) for species “Homo sapiens.” The listed pathways were curated by submitting down-regulated proteins altered with LPS-Cytokines and presented in Table 1.
