# Supplementary material for: A multi-mineral intervention to counter pro-inflammatory activity and to improve the barrier in human colon organoids
Source: Front Cell Dev Biol. 2023 Jul 5;11:1132905. doi: 10.3389/fcell.2023.1132905 (PMC10354648; doi:10.3389/fcell.2023.1132905)
Supplement: Supplementary file 1 [file DataSheet1.zip › Supplementary Table S9.PDF]

Supplement Table 9. Pathways associated with up-regulated proteins altered with Aquamin in the presence of LPS-Cytokines.

Pathways	Entities pValue	Mapped entities
Interferon Signaling	$1.11x10^{-16}$	HLA-E;HLA-A;TRIM21;OAS3;HLA-G;PML;HLA-DRB4;EIF2AK2;STAT1;HLA-DRB1;HLA-DRB3;HLA-DPB1;B2M; HLA-DPA1;IFITM2;HLA-F;DDX58;MX1;GBP1;GBP2;HLA-C;IFI30;HLA-DRA;ISG15; HLA-B;ICAM1;OAS1;UBE2L6;UBA7;HLA-DRB5
Interferon gamma signaling	$1.11x10^{-16}$	HLA-F;HLA-E;HLA-A;TRIM21;OAS3;HLA-G;PML;GBP1;GBP2;HLA-DRB4;HLA-C;STAT1;IFI30;HLA-DRA; HLA-DRB1;HLA-B;HLA-DRB3;ICAM1;OAS1;HLA-DPB1;B2M;HLA-DPA1;HLA-DRB5
Cytokine Signaling in Immune system	$1.11x10^{-16}$	IL32;HLA-E;HLA-A;PSMB9;TRIM21;OAS3;HLA-G;PML;GSDMD;PSME1;MUC1;HLA-DRB4;EIF2AK2;STAT1; HLA-DRB1;HLA-DRB3;CD40;PSMB10;HLA-DPB1;B2M;HLA-DPA1;IFITM2;HLA-F;DDX58;PSME2;CASP1; MX1;GBP1;NOS2;GBP2;HLA-C;IFI30;HLA-DRA;ISG15;HLA-B;ICAM1;SERPINB2;OAS1;UBE2L6;UBA7; HLA-DRB5
Immune System	$5.93x10^{-14}$	IL32;IFI16;HLA-A;HLA-E;OAS3;GSDMD;EIF2AK2;TMBIM1;STAT1;HLA-DRB1;HLA-DRB3;HLA-DPB1; HLA-DPA1;MUC17;HLA-F;CASP1;HRNR;GBP1;RNF213;GBP2;BTN3A3;HLA-C;ANPEP;IFI30;CD55; HLA-DRA;OLFM4;FBXO6;OAS1;HLA-DMB;PSMB9;TRIM21;HLA-G;PML;PSME1;MUC1;HLA-DRB4; TAPBP;CD40;PSMB10;KRT1;B2M;IFITM2;DDX58;PSME2;MX1;NOS2;CTSV;S100A7;ISG15;FLG2; SERPINA1;HLA-B;ICAM1;SERPINB2;TAP1;CFB;TAP2;DCD;UBA7;CD74;UBE2L6;HLA-DRB5;LGMN
Interferon alpha/beta signaling	$2.16x10^{-10}$	HLA-F;MX1;HLA-E;HLA-A;HLA-G;OAS3;HLA-C;GBP2;STAT1;ISG15;HLA-B;OAS1;IFITM2
Antigen processing-Cross presentation	$3.85x10^{-9}$	HLA-F;PSME2;HLA-E;HLA-A;PSMB9;HLA-G;PSME1;CTSV;HLA-C;HLA-B;TAPBP;PSMB10;B2M;TAP1;TAP2
Adaptive Immune System	$5.75x10^{-9}$	HLA-DMB;HLA-E;HLA-A;PSMB9;TRIM21;HLA-G;PSME1;HLA-DRB4;HLA-DRB1;HLA-DRB3;TAPBP;CD40;PSMB10; HLA-DPB1;B2M;HLA-DPA1;HLA-F;PSME2;RNF213;CTSV;HLA-C;BTN3A3;IFI30;HLA-DRA;HLA-B;FBXO6; ICAM1;TAP1;TAP2;CD74;UBE2L6;UBA7;HLA-DRB5;LGMN
ER-Phagosome pathway	$9.46x10^{-9}$	HLA-F;PSME2;HLA-E;HLA-A;PSMB9;HLA-G;PSME1;HLA-C;HLA-B;TAPBP;PSMB10;B2M;TAP1;TAP2
MHC class II antigen presentation	$1.64x10^{-7}$	HLA-DMB;IFI30;HLA-DRA;HLA-DRB1;HLA-DRB3;HLA-DPB1;CD74;CTSV;HLA-DPA1;HLA-DRB4;LGMN;HLA-DRB5
Downstream TCR signaling	$1.78x10^{-7}$	PSME2;HLA-DRA;PSMB9;HLA-DRB1;HLA-DRB3;PSMB10;PSME1;HLA-DPB1;HLA-DPA1;HLA-DRB4;HLA-DRB5
Antigen Presentation: Folding, assembly and peptide loading of class I MHC	$3.39x10^{-7}$	HLA-F;HLA-A;HLA-E;HLA-B;TAPBP;HLA-G;B2M;TAP1;TAP2;HLA-C
Translocation of ZAP-70 to Immunological synapse	$4.48x10^{-7}$	HLA-DRA;HLA-DRB1;HLA-DRB3;HLA-DPB1;HLA-DPA1;HLA-DRB5;HLA-DRB4
Class I MHC mediated antigen processing & presentation	$4.76x10^{-7}$	HLA-F;PSME2;HLA-E;HLA-A;PSMB9;TRIM21;HLA-G;PSME1;RNF213;CTSV;HLA-C; HLA-B;TAPBP;FBXO6;PSMB10;B2M;TAP1;TAP2;UBA7;UBE2L6
Phosphorylation of CD3 and TCR zeta chains	$7.40x10^{-7}$	HLA-DRA;HLA-DRB1;HLA-DRB3;HLA-DPB1;HLA-DPA1;HLA-DRB5;HLA-DRB4
PD-1 signaling	$8.67x10^{-7}$	HLA-DRA;HLA-DRB1;HLA-DRB3;HLA-DPB1;HLA-DPA1;HLA-DRB5;HLA-DRB4
TCR signaling	$9.62x10^{-7}$	PSME2;HLA-DRA;PSMB9;HLA-DRB1;HLA-DRB3;PSMB10;PSME1;HLA-DPB1;HLA-DPA1;HLA-DRB4;HLA-DRB5
Antiviral mechanism by IFN-stimulated genes	$1.44x10^{-6}$	EIF2AK2;DDX58;STAT1;MX1;ISG15;OAS3;OAS1;UBA7;UBE2L6
Generation of second messenger molecules	$3.96x10^{-6}$	HLA-DRA;HLA-DRB1;HLA-DRB3;HLA-DPB1;HLA-DPA1;HLA-DRB5;HLA-DRB4
Endosomal/Vacuolar pathway	$5.55x10^{-6}$	HLA-F;HLA-A;HLA-E;HLA-B;HLA-G;B2M;CTSV;HLA-C
SARS-CoV-2 activates/modulates innate and adaptive immune responses	$9.49x10^{-6}$	HLA-F;DDX58;STAT1;HLA-A;HLA-E;ISG15;HLA-B;HLA-G;B2M;HLA-C
Innate Immune System	$9.95x10^{-6}$	IFI16;HLA-E;PSMB9;TRIM21;GSDMD;PSME1;MUC1;TMBIM1;PSMB10;KRT1;B2M;MUC17;DDX58;PSME2;CASP1; HRNR;NOS2;CTSV;HLA-C;ANPEP;CD55;S100A7;ISG15;FLG2;SERPINA1;HLA-B;OLFM4;CFB;DCD;UBE2L6; UBA7;LGMN
ISG15 antiviral mechanism	$5.43x10^{-5}$	EIF2AK2;DDX58;STAT1;MX1;ISG15;UBA7;UBE2L6

Costimulation by the CD28 family	$1.12x10^{-4}$	HLA-DRA;HLA-DRB1;HLA-DRB3;HLA-DPB1;HLA-DPA1;HLA-DRB5;HLA-DRB4
SARS-CoV-2-host interactions	$1.83x10^{-4}$	HLA-F;DDX58;STAT1;HLA-A;HLA-E;ISG15;HLA-B;HLA-G;B2M;HLA-C
OAS antiviral response	$2.51x10^{-4}$	DDX58;OAS3;OAS1
Loss of MECP2 binding ability to 5hmC-DNA	$2.76x10^{-4}$	MECP2
SARS-CoV-2 Infection	$5.42x10^{-4}$	HLA-F;DDX58;STAT1;HLA-E;HLA-A;ISG15;HLA-B;HLA-G;PARP9;B2M;HLA-C
Negative regulators of DDX58/IFIH1 signaling	$8.65x10^{-4}$	DDX58;ISG15;UBE2L6;UBA7
MECP2 regulates transcription of genes involved in GABA signaling	0.001	MECP2
Loss of MECP2 binding ability to 5mC-DNA	0.001	MECP2
KEAP1-NFE2L2 pathway	0.002	PSME2;GSTA1;PSMB9;TRIM21;PSMB10;PSME1
Loss of phosphorylation of MECP2 at T308	0.002	MECP2
Fructose catabolism	0.002	ALDOB;ALDH1A1
Regulation of RUNX2 expression and activity	0.002	STAT1;PSME2;PSMB9;PSMB10;PSME1
MECP2 regulates transcription factors	0.002	MECP2
Nuclear events mediated by NFE2L2	0.003	PSME2;GSTA1;PSMB9;PSMB10;PSME1
Regulation of activated PAK-2p34 by proteasome mediated degradation	0.003	PSME2;PSMB9;PSMB10;PSME1
Cross-presentation of soluble exogenous antigens (endosomes)	0.003	PSME2;PSMB9;PSMB10;PSME1
Fructose metabolism	0.003	ALDOB;ALDH1A1
Regulation of ornithine decarboxylase (ODC)	0.003	PSME2;PSMB9;PSMB10;PSME1
p53-Independent DNA Damage Response	0.004	PSME2;PSMB9;PSMB10;PSME1
Ubiquitin Mediated Degradation of Phosphorylated Cdc25A	0.004	PSME2;PSMB9;PSMB10;PSME1
p53-Independent G1/S DNA damage checkpoint	0.004	PSME2;PSMB9;PSMB10;PSME1
GSK3B and BTRC:CUL1-mediated-degradation of NFE2L2	0.004	PSME2;PSMB9;PSMB10;PSME1
Autodegradation of the E3 ubiquitin ligase COP1	0.004	PSME2;PSMB9;PSMB10;PSME1
Ubiquitin-dependent degradation of Cyclin D	0.004	PSME2;PSMB9;PSMB10;PSME1
Vpu mediated degradation of CD4	0.004	PSME2;PSMB9;PSMB10;PSME1
Regulation of Apoptosis	0.004	PSME2;PSMB9;PSMB10;PSME1
Signaling by Interleukins	0.004	STAT1;PSME2;CASP1;IL32;PSMB9;ICAM1;SERPINB2;PSMB10;GSDMD;PSME1;MUC1;NOS2
Loss of MECP2 binding ability to the NCoR/SMRT complex	0.004	MECP2
FBXL7 down-regulates AURKA during mitotic entry and in early mitosis	0.004	PSME2;PSMB9;PSMB10;PSME1
SCF-beta-TrCP mediated degradation of Emi1	0.004	PSME2;PSMB9;PSMB10;PSME1
Degradation of AXIN	0.004	PSME2;PSMB9;PSMB10;PSME1

Negative regulation of NOTCH4 signaling	0.004	PSME2;PSMB9;PSMB10;PSME1
Regulation of RUNX3 expression and activity	0.004	PSME2;PSMB9;PSMB10;PSME1
Programmed Cell Death	0.005	DSG2;PSME2;CASP1;CASP7;PSMB9;PSMB10;GSDMD;PSME1
Formation of the cornified envelope	0.005	DSG2;KRT16;KRT9;KRT2;KRT6A;KRT1
AUF1 (hnRNP D0) binds and destabilizes mRNA	0.005	PSME2;PSMB9;PSMB10;PSME1
Vif-mediated degradation of APOBEC3G	0.005	PSME2;PSMB9;PSMB10;PSME1
Hh mutants are degraded by ERAD	0.005	PSME2;PSMB9;PSMB10;PSME1
Neutrophil degranulation	0.005	HRNR;GSDMD;HLA-C;TMBIM1;ANPEP;CD55;S100A7;FLG2;OLFM4;SERPINA1;HLA-B;B2M;KRT1
Degradation of DVL	0.005	PSME2;PSMB9;PSMB10;PSME1
Stabilization of p53	0.005	PSME2;PSMB9;PSMB10;PSME1
MECP2 regulates transcription of neuronal ligands	0.005	MECP2
Interleukin-1 processing	0.005	CASP1;GSDMD
Hh mutants abrogate ligand secretion	0.006	PSME2;PSMB9;PSMB10;PSME1
NIK-->noncanonical NF-kB signaling	0.006	PSME2;PSMB9;PSMB10;PSME1
Metabolism of polyamines	0.006	PSME2;PSMB9;PSMB10;PSME1
Degradation of GLI1 by the proteasome	0.006	PSME2;PSMB9;PSMB10;PSME1
GLI3 is processed to GLI3R by the proteasome	0.006	PSME2;PSMB9;PSMB10;PSME1
Degradation of GLI2 by the proteasome	0.006	PSME2;PSMB9;PSMB10;PSME1
SCF(Skp2)-mediated degradation of p27/p21	0.006	PSME2;PSMB9;PSMB10;PSME1
Dectin-1 mediated noncanonical NF-kB signaling	0.006	PSME2;PSMB9;PSMB10;PSME1
Defective CFTR causes cystic fibrosis	0.006	PSME2;PSMB9;PSMB10;PSME1
Apoptosis	0.006	DSG2;PSME2;CASP7;PSMB9;PSMB10;GSDMD;PSME1
Termination of translesion DNA synthesis	0.007	ISG15;UBE2L6;UBA7
Autodegradation of Cdh1 by Cdh1:APC/C	0.007	PSME2;PSMB9;PSMB10;PSME1
Asymmetric localization of PCP proteins	0.007	PSME2;PSMB9;PSMB10;PSME1
TNFR2 non-canonical NF-kB pathway	0.008	PSME2;PSMB9;CD40;PSMB10;PSME1
Metallothioneins bind metals	0.008	MT1E;MT1H
Hedgehog ligand biogenesis	0.008	PSME2;PSMB9;PSMB10;PSME1
Oxygen-dependent proline hydroxylation of Hypoxia-inducible Factor Alpha	0.008	PSME2;PSMB9;PSMB10;PSME1
p53-Dependent G1 DNA Damage Response	0.008	PSME2;PSMB9;PSMB10;PSME1
p53-Dependent G1/S DNA damage checkpoint	0.008	PSME2;PSMB9;PSMB10;PSME1
Activation of NF-kappaB in B cells	0.009	PSME2;PSMB9;PSMB10;PSME1
APC/C:Cdc20 mediated degradation of Securin	0.009	PSME2;PSMB9;PSMB10;PSME1

G1/S DNA Damage Checkpoints	0.009	PSME2;PSMB9;PSMB10;PSME1
Immunoregulatory interactions between a Lymphoid and a non-Lymphoid cell	0.009	HLA-F;HLA-A;HLA-E;HLA-B;CD40;ICAM1;HLA-G;B2M;HLA-C
Regulation of RAS by GAPs	0.010	PSME2;PSMB9;PSMB10;PSME1
Regulation of PTEN stability and activity	0.010	PSME2;PSMB9;PSMB10;PSME1
Orc1 removal from chromatin	0.011	PSME2;PSMB9;PSMB10;PSME1
Disorders of Developmental Biology	0.011	MECP2
Pervasive developmental disorders	0.011	MECP2
Loss of function of MECP2 in Rett syndrome	0.011	MECP2
Disorders of Nervous System Development	0.011	MECP2
Trafficking and processing of endosomal TLR	0.011	CTSV;LGMN
Interleukin-1 family signaling	0.011	PSME2;CASP1;PSMB9;PSMB10;GSDMD;PSME1
Translesion synthesis by Y family DNA polymerases bypasses lesions on DNA template	0.012	ISG15;UBE2L6;UBA7
Cdc20:Phospho-APC/C mediated degradation of Cyclin A	0.012	PSME2;PSMB9;PSMB10;PSME1
CDK-mediated phosphorylation and removal of Cdc6	0.012	PSME2;PSMB9;PSMB10;PSME1
SARS-CoV Infections	0.012	HLA-F;DDX58;STAT1;HLA-E;HLA-A;ISG15;HLA-B;HLA-G;PARP9;B2M;HLA-C
Hereditary fructose intolerance	0.012	ALDOB
Defective SLC11A2 causes hypochromic microcytic anemia, with iron overload 1 (AHMIO1)	0.012	SLC11A2
Antigen processing: Ubiquitination & Proteasome degradation	0.012	PSME2;PSMB9;FBXO6;TRIM21;PSMB10;PSME1;RNF213;UBA7;UBE2L6
Signaling by NOTCH	0.012	STAT1;PSME2;PLXND1;PSMB9;PSMB10;PSME1;MDK
APC/C:Cdh1 mediated degradation of Cdc20 and other APC/C:Cdh1 targeted proteins in late mitosis/early G1	0.012	PSME2;PSMB9;PSMB10;PSME1
APC:Cdc20 mediated degradation of cell cycle proteins prior to satisfation of the cell cycle checkpoint	0.012	PSME2;PSMB9;PSMB10;PSME1
Response to metal ions	0.012	MT1E;MT1H
APC/C:Cdc20 mediated degradation of mitotic proteins	0.013	PSME2;PSMB9;PSMB10;PSME1
Cellular response to hypoxia	0.013	PSME2;PSMB9;PSMB10;PSME1
Activation of APC/C and APC/C:Cdc20 mediated degradation of mitotic proteins	0.014	PSME2;PSMB9;PSMB10;PSME1
Interleukin-6 signaling	0.014	STAT1
ABC transporter disorders	0.014	PSME2;PSMB9;PSMB10;PSME1

The role of GTSE1 in G2/M progression after G2 checkpoint	0.015	PSME2;PSMB9;PSMB10;PSME1
Transcriptional regulation by RUNX2	0.015	STAT1;PSME2;PSMB9;PSMB10;PSME1
STING mediated induction of host immune responses	0.016	IFI16;TRIM21
Regulation of APC/C activators between G1/S and early anaphase	0.016	PSME2;PSMB9;PSMB10;PSME1
Downstream signaling events of B Cell Receptor (BCR)	0.017	PSME2;PSMB9;PSMB10;PSME1
Cyclin E associated events during G1/S transition	0.018	PSME2;PSMB9;PSMB10;PSME1
Signaling by NOTCH4	0.018	PSME2;PSMB9;PSMB10;PSME1
Degradation of beta-catenin by the destruction complex	0.018	PSME2;PSMB9;PSMB10;PSME1
C-type lectin receptors (CLRs)	0.018	MUC17;PSME2;PSMB9;PSMB10;PSME1;MUC1
DDX58/IFIH1-mediated induction of interferon-alpha/beta	0.019	DDX58;ISG15;UBE2L6;UBA7
Hedgehog 'on' state	0.019	PSME2;PSMB9;PSMB10;PSME1
Cyclin A:Cdk2-associated events at S phase entry	0.019	PSME2;PSMB9;PSMB10;PSME1
MECP2 regulates neuronal receptors and channels	0.020	MECP2
Signaling by cytosolic FGFR1 fusion mutants	0.020	STAT1;ZMYM2
DNA Damage Bypass	0.020	ISG15;UBE2L6;UBA7
APC/C-mediated degradation of cell cycle proteins	0.021	PSME2;PSMB9;PSMB10;PSME1
Regulation of mitotic cell cycle	0.021	PSME2;PSMB9;PSMB10;PSME1
Regulation of mRNA stability by proteins that bind AU-rich elements	0.021	PSME2;PSMB9;PSMB10;PSME1
Disorders of transmembrane transporters	0.022	CP;PSME2;PSMB9;PSMB10;PSME1;SLC11A2
Defective GALNT3 causes HFTC	0.022	MUC17;MUC1
Defective GALNT12 causes CRCS1	0.022	MUC17;MUC1
MAPK6/MAPK4 signaling	0.022	PSME2;PSMB9;PSMB10;PSME1
Defective SLC40A1 causes hemochromatosis 4 (HFE4) (macrophages)	0.023	CP
Defective CP causes aceruloplasminemia (ACERULOP)	0.023	CP
Inhibition of PKR	0.023	EIF2AK2
Defective C1GALT1C1 causes TNPS	0.024	MUC17;MUC1
Aflatoxin activation and detoxification	0.024	CYP3A4;ACY3

Nuclear events stimulated by ALK signaling in cancer	0.024	MECP2
Apoptotic factor-mediated response	0.024	CASP7;GSDMD
Switching of origins to a post-replicative state	0.025	PSME2;PSMB9;PSMB10;PSME1
PCP/CE pathway	0.025	PSME2;PSMB9;PSMB10;PSME1
PTEN Regulation	0.026	PSME2;PSMB9;PML;PSMB10;PSME1
UCH proteinases	0.028	PSME2;PSMB9;PSMB10;PSME1
Transcriptional regulation by RUNX3	0.028	PSME2;PSMB9;PSMB10;PSME1
Signaling by phosphorylated juxtamembrane, extracellular and kinase domain KIT mutants	0.029	STAT1
Signaling by KIT in disease	0.029	STAT1
Host Interactions of HIV factors	0.029	PSME2;PSMB9;PSMB10;PSME1;B2M
Interleukin-12 family signaling	0.030	STAT1;PSME2;SERPINB2
CLEC7A (Dectin-1) signaling	0.030	PSME2;PSMB9;PSMB10;PSME1
Cellular response to chemical stress	0.030	PSME2;GSTA1;PSMB9;TRIM21;PSMB10;PSME1
Signaling by ALK fusions and activated point mutants	0.031	MECP2;RNF213
Signaling by ALK in cancer	0.031	MECP2;RNF213
RUNX1 regulates transcription of genes involved in differentiation of HSCs	0.031	PSME2;PSMB9;PSMB10;PSME1
The AIM2 inflammasome	0.035	CASP1
ABC-family proteins mediated transport	0.035	PSME2;PSMB9;PSMB10;PSME1
NOTCH3 Intracellular Domain Regulates Transcription	0.036	STAT1;PLXND1
Transcriptional regulation by RUNX1	0.039	PSME2;PSMB9;PML;PSMB10;PSME1;CTSV
Termination of O-glycan biosynthesis	0.039	MUC17;MUC1
Regulation of IFNA/IFNB signaling	0.039	STAT1
Metal ion SLC transporters	0.039	CP;SLC11A2
Pyroptosis	0.041	CASP1;GSDMD
Cell recruitment (pro-inflammatory response)	0.041	CASP1;GSDMD
Purinergic signaling in leishmaniasis infection	0.041	CASP1;GSDMD
Interleukin-4 and Interleukin-13 signaling	0.044	STAT1;ICAM1;NOS2;MUC1
Interleukin-6 family signaling	0.044	STAT1
Assembly of the pre-replicative complex	0.045	PSME2;PSMB9;PSMB10;PSME1
Keratinization	0.046	DSG2;KRT16;KRT9;KRT2;KRT6A;KRT1
Inhibition of nitric oxide production	0.046	NOS2

Diseases of signal transduction by growth factor receptors and second messengers	0.047	STAT1;PSME2;ZMYM2;PSMB9;MECP2;PSMB10;PSME1;RNF213
Hedgehog 'off' state	0.048	PSME2;PSMB9;PSMB10;PSME1
Interleukin-1 signaling	0.049	PSME2;PSMB9;PSMB10;PSME1
DAP12 signaling	0.050	HLA-E;B2M

The pathway analysis report was created by employing Reactome pathway database (v82) for species “Homo sapiens.” The listed pathways were curated by submitting up-regulated proteins altered with LPS-Cytokines in the presence of Aquamin.
